# ^64^Cu Hypoxia Imaging Radiotracer Targeting the Human Copper Transporter

**DOI:** 10.1007/s12017-026-08939-4

**Published:** 2026-06-29

**Authors:** Shelly Meron, Yulia Shenberger, Ravit Madar, Jana Aupic, Nathalie Abudi, Fabio Lapenta, Melanie Hirsch, Odelia Orbaum Harel, Lukas Hofmann, Alessandra Magistrato, Eitan Okun, Rinat Abramovitch, Sharon Ruthstein

**Affiliations:** 1 Department of Chemistry, Institute of Nanotechnology and Advanced Materials, Faculty of Exact Sciences, Bar-Ilan University, Ramat-Gan, 5290002 Israel; 2 The Mina and Everard Goodman Faculty of Life Sciences, Bar-Ilan University, Ramat Gan, 5290002 Israel; 3 The Gonda Multidisciplinary Brain Research Center, Bar-Ilan University, Ramat Gan, 5290002 Israel; 4 The Paul Feder Laboratory for Alzheimer’s disease research, Bar-Ilan University, Ramat Gan, 5290002 Israel; 5 CNR-IOM at SISSA, Trieste, 34135 Italy; 6 Wohl Institute for Translational Medicine, Hadassah Medical Center, Jerusalem, 91120 Israel; 7 Faculty of Medicine, Hebrew University of Jerusalem, Jerusalem, 91904 Israel; 8 Laboratory for Environmental and Life Sciences, University of Nova Gorica, Nova Gorica, 5000 Slovenia

**Keywords:** Hypoxia, ^64^Cu, hCtr1 transporter, PET-MRI, Radiopharmaceuticals

## Abstract

The detection and characterization of hypoxia through functional imaging represents a critical frontier in oncology, cardiology, and neurology. Despite extensive efforts, the clinical translation of a robust hypoxia imaging biomarker remains hindered by suboptimal signal limiting diagnostic accuracy. Here, we report the development of a ^64^Cu-based radiotracer (^64^Cu-CysPhe), which exploits the cellular copper uptake pathway involving the human copper transporter 1 (hCtr1). This targeted mechanism facilitates selective tracer accumulation in hypoxic tissues. In-vivo PET-MRI imaging in a murine breast cancer model demonstrated tumor-to-muscle ratios exceeding 3.0 up to 24 h post-injection. Notably, immunohistochemical analyses revealed that ^64^Cu-CysPhe preferentially localizes to hypoxic tumor regions while excluding necrotic cores, thereby providing information about a tumor’s immediate microenvironment. Comparative analysis with established radiotracers, including ^64^Cu-ATSM and ^18^F-FDG, confirmed the sensitivity of ^64^Cu-CysPhe in detecting hypoxic tumor regions. These findings establish ^64^Cu-CysPhe as a promising candidate for non-invasive imaging of tumors.

**Supplementary Information:**

The online version contains supplementary material available at 10.1007/s12017-026-08939-4.

## Introduction

Hypoxia is a physiological condition characterized by an insufficient oxygen supply required to maintain tissue homeostasis. The ability to accurately and reliably image hypoxia is of critical importance across various medical fields, including cardiology, neurology, and oncology (Hockel et al. 1996a, b; Mees et al. 2009; Park et al. 2015; Raz et al. 2015; Soon et al. 2011; Sun et al. 2010; Syme et al. 2004; Wilson and Hay 2011). Within the tumor microenvironment, hypoxia plays a pivotal role in promoting cellular proliferation, angiogenesis, and resistance to therapy (Hockel et al. 1996a, b; Kizaka-Kondoh and Konse-Nagasawa 2009; Kuo and Le 2014; Lehtiο et al., 2001; Rajendran et al., 2006; Vaupel & Mayer, 2007). Despite extensive efforts to develop reliable imaging modalities for hypoxia detection, no clinically approved hypoxia-specific biomarker has yet been incorporated into routine practice. This limitation stems from both low tumor uptake and a sub-optimal tumor-to-background ratio, which compromises imaging quality. In this study, we present a new radiotracer designed to overcome these limitations, offering improved detection of hypoxic conditions in tumors in-vivo.

Molecular imaging, particularly X-ray computed tomography (CT) and magnetic resonance imaging (MRI), have long been the gold standards for accurate localization of organs and lesions in radiation oncology. However, the effectiveness of such structural imaging techniques in determining metabolic or functional tissue information is limited. Positron emission tomography (PET), single photon emission computed tomography (SPECT), as well as dual imaging modalities including PET/CT, and PET/MRI offer significantly enhanced imaging capabilities, enabling visualization of a wide range of physiological phenomena (Anderson & Ferdani, 2009; Arman & Habib, 2008; Millar et al., 2013; Szyszko et al., 2016; Wernick, 2004), due to the selection of radiotracers that specifically target particular physiological mechanisms or molecular targets. Radiotracers are examples of radiopharmaceuticals, i.e., compounds labeled with radionuclides that emit ionizing radiation and are extensively utilized in nuclear imaging (in combination with CT or MRI) for both diagnostic and therapeutic purposes. Given the continuous rise in cancer incidence (Rosenberg & Miranda-Filho, 2024), there is an urgent demand for novel radiotracers capable of facilitating early diagnosis. Currently available radiotracers, such as ^18^F-2-fluoro-2-deoxy-glucose (^18^F-FDG), primarily reflect glucose metabolism (Hansen et al. 2012b; Liu et al. 2009; Millar et al. 2013), as heightened glucose consumption is a hallmark of many cancers. However, increased glucose uptake is not exclusive to malignant cells. Moreover, not all tumors consume glucose, including carcinoid, prostate, head and neck tumors (Shokeen & Anderson, 2008; Vavere & Lewis, 2007). This limitation highlights the need for alternative imaging approaches that do not rely on glucose metabolism, such as those that target hypoxia (McConathy & Sheline, 2015; Xie et al., 2016). Indeed, approximately 90% of all solid tumors exhibit hypoxic regions or a hypoxic core (Chen et al., 2023). The development of such radiotracers will enhance diagnostic accuracy and potentially guide personalized treatment strategies in oncology.

In an effort to develop accurate and non-invasive techniques for assessing tumor hypoxia, several PET radiotracers have been synthesized, including various nitroimidazole derivatives, such as ¹⁸F-fluoromisonidazole ([^18^F]-FMISO) (Liu et al., 2009; Zheng et al., 2015). The mechanism of action of these biomarkers based on the reduction of the nitrogen dioxide group to an amine in low-oxygen environments. This amine complex is subsequently trapped within the cellular cycle by various proteins, leading to increased retention of the tracer in hypoxic tissues, as compared to normoxic tissues (Barajas et al., 2022; Carles et al., 2021; Dos Santos et al., 2023). However, these tracers are not routinely used due to their slow and passive cellular uptake (Bruycker et al., 2016; Szyszko et al., 2016; Wang et al., 2016; Wei et al., 2016; Zhang et al., 2016). Consequently, a post-injection waiting period of approximately 2–3 h is required to achieve a sufficient tumor-to-background ratio, a timeframe approaching and exceeding the half-life of the ¹⁸F isotope (109.7 min). As a result, higher exposure to radioactive material is often necessary, such that, under some conditions, to improve the sensitivity of detection (Bruycker et al., 2016; Postema et al., 2009; Szyszko et al., 2016; Valable et al., 2011; Wang et al., 2016; Wei et al., 2016; Zhang et al., 2016). Of late, researchers have increasingly focused on metal-based radiotracers to enhance the sensitivity of cellular oxygen pressure measurements, given the redox potential of certain metals. Copper has attracted significant attention due to its biological relevance. As an essential trace element in humans, copper serves as a cofactor for numerous enzymes and proteins critical for respiration, iron transport, metabolism, cell growth, and homeostasis (Syme et al., 2004; Uriu-Adams & Keen, 2005; Wernimont et al., 2000). In recent years, copper isotopes conjugated to antibodies, proteins, peptides, and nanoparticles have found increasing use in pre-clinical and clinical research for the detection and treatment of pathological conditions affecting copper metabolism (Asabella et al., 2014; Cooper et al., 2012; Martin et al., 2021; Nagle et al., 2021; Nomura et al., 2014). A number of animal and human studies have investigated the efficacy of radiotracers based on ^64^Cu(II) to detect hypoxia (Asabella et al., 2014), such as ^64^Cu(II)-diacetyl-bis(*N*^4^-methylthiosemicarbazone) (^64^Cu-ATSM), these have been subsequently proposed as promising PET imaging agents for hypoxia assessment (Liu et al., 2021; Vavere & Lewis, 2007; Wadas et al., 2010; Xie et al., 2016; Zeglis et al., 2014). ^64^Cu isotope labeling offers practical advantages for imaging due to its half-life of 12.7 h, sufficiently long for handling and imaging procedures, while minimizing excessive patient radiation exposure. The clinical relevance of ^64^Cu-ATSM in assessing hypoxia in tumors has been validated in multiple studies, where the potential to predict tumor behavior and treatment response was demonstrated (Hansen et al. 2012a; Laforest et al. 2005; Park et al. 2015; Soon et al. 2011; Vavere and Lewis 2007). Notably, while imaging with ^18^F-FDG is accompanied by limitations in distinguishing benign from malignant tissues, ^64^Cu-ATSM has demonstrated superior specificity in identifying hypoxic tissues (Colombie et al., 2015). However, despite extensive research, this tracer has yet to achieve widespread clinical use. One of the primary limitations of ^64^Cu-ATSM is the incomplete understanding of its cellular mechanism of action. Although ^64^Cu-ATSM was suggested to enter cells *via* both passive diffusion across the cellular membrane and active transport by the human copper transporter 1 (hCtr1, also known as SLC31A1), reliance on this dual mechanism has led to irreproducible results (Colombie et al., 2015). Interestingly, over-expression of hCtr1 has been observed in various cancers, including melanoma, breast, liver, lung, and prostate cancers (Blockhuys et al., 2021; Zhang et al., 2022, 2023), leading to elevated copper accumulation in malignant tissues. Therefore, targeting hCtr1 may enhance the selectivity of ^64^Cu-based radiotracers for tumor cells, potentially improving their diagnostic and therapeutic efficacy.

Here, we describe the design of a novel compound, ^64^Cu-CysPhe, which consists of a peptide, a ligand, and a radionuclide copper ion with high affinity for hCtr1. In-vitro experiments demonstrated that ^64^Cu-CysPhe uptake may involve hCtr1 and is influenced by the hypoxic condition of the cell. Furthermore, in-vivo studies in a breast cancer mouse model revealed that ^64^Cu-CysPhe exhibits high sensitivity in reporting hypoxic tumor regions, which will potentially assist in diagnosing hypoxic conditions in tumors and other tissues.

## Results

### ^64^Cu-CysPhe Design

**Fig. 1:**
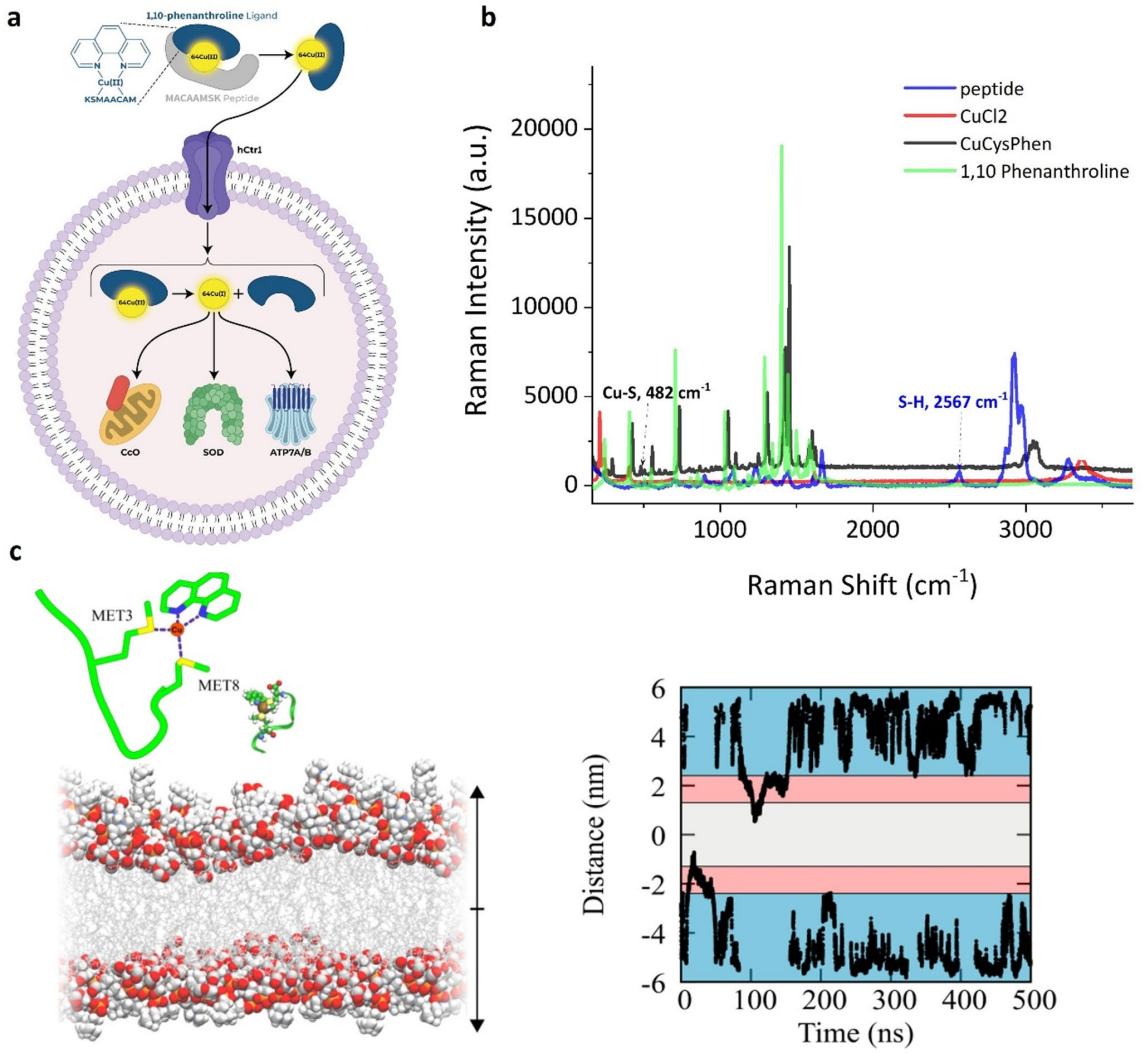
**a**. Schematic representation of the mechanism of action of ^64^Cu-CysPhe. Under hypoxic conditions, with low pH and a reduced cellular environment, Cu(II) is reduced to Cu(I), which prolongs Cu retention time in the cell, and ligand dissociation from the complex. **b**. Raman spectroscopy measurements confirmed coordination of Cu(II) ion with the ligand and peptide via sulfur atoms of the peptide and nitrogen atoms of the ligand. **c**. All-atom simulations. The left part of the simulation shows the 3D structure of Cu-CysPhe as displayed in the representative frame of the all-atom metadynamics simulation. Cu-CysPhe is shown in green. Cu-CysPhe approaches a model of a membrane assembled by POPC. The lipid polar head groups are shown as van der Waals spheres, while hydrophobic lipid chains are shown as grey lines. The arrows denote the collective variable (CV) used in the metadynamics simulations. The right part shows the distance between the center of mass of the POPC membrane and Cu-CysPhe along the Z axis. The area of the simulation box corresponding to the aqueous solution is colored blue, while the polar heads and hydrophobic acyl chains of the lipid bilayer are marked in pink and grey, respectively. Cu-CysPhe could not pass the membrane

The copper transporter hCtr1 relies on its extracellular N-terminal domain to accept copper in the Cu(II) oxidation state from blood carrier proteins (Shenberger et al., 2015). It then reduces Cu(II) to Cu(I) via a still unresolved mechanism. (Janos et al., 2022; Magistrato et al., 2019; Shenberger et al., 2015; Stefaniak et al., 2018; Walke et al., 2022). Subsequent intracellular distribution of Cu(I) is mediated by various metallochaperones. To enable efficient Cu(II) and Cu(I) binding, the extracellular domain of hCtr1 relies on several histidine and methionine residues. The two Cu(II)-binding sites rely on ^1^MDHSHH and ^22^HHH residues, while the ^7^MGMSYM and ^41^MMMPM motifs (Met motifs) serve as the first Cu(I)-binding sites of hCtr1. Bound Cu(I) are then transformed to other Met motifs in the transmembrane and intracellular regions of the protein (Magistrato et al., 2019; Peleg et al., 2025; Shenberger et al., 2015; Walke et al., 2022). To ensure binding to hCtr1 and possible transfer through the various Cu(I)-binding sites, we designed a compound comprising three components, namely, a ligand, Cu(II), and a peptide. The peptide serves to guide the Cu(II) complex to an hCtr1 Met motif before dissociating upon binding (Fig. 1a). This design is inspired by a gear-like mechanism that mimics Cu(I) transfer between Met motifs. The peptide KSMAACAM (termed Cys) contains two Met residues (**Figure ****S1**,** SI**). Cu(II) coordination typically requires 4–6 coordinating atoms, often involving His, Cys, or Met residues (Aronoff-Spencer et al., 2000; Jun & Saxena, 2007), whereas Cu(I) requires only 2–3 ligands, usually Met or Cys residues (Robinson & Winge, 2010; Rubino et al., 2010). To complete the coordination sphere of Cu(II), the organic ligand 1,10-phenanthroline (Phe) was introduced, providing two additional coordination sites. The ligand Phe serves a dual role, namely, precluding passive transport and thus favoring the formation of the Cu(II)-Cys-Phe complex at the hCtr1 Met motif, after dissociation of the Cys peptide, and stabilizing the Cu(II) oxidation state under normoxic conditions. In a hypoxic environment, the Phe ligand is protonated under the reduced pH of the hypoxic environment, leading to dissociation of Cu(II), that is subsequently reduced to Cu(I). Cu(I) exhibits a longer cellular retention time than does the Cu(II)-Phe complex as cellular proteins hold higher affinity for Cu(I). Accordingly, under hypoxic conditions, increased uptake and enhanced tumor detectability are anticipated, relative to normoxic conditions.

Formation of a Cu-CysPhe complex was verified by Raman (Fig. 1b) and electrospray ionization mass spectrometry (ESI-MS) (**Figure S2**,** SI**). ESI-MS analysis under soft ionization conditions revealed that, in the presence of Cu(II) ions, non-covalent complexes formed with both the peptide and the ligand. Raman analysis of the non-radioactive Cu-CysPhe crystallized compound suggested Cu-S coordination. Disappearance of the peak associated with the S-H stretch at 2567 cm^− 1^ (Minkov et al., 2010), indicative of deprotonation and subsequent coordination to Cu, along with the emergence of a new peak at 482 cm⁻¹, corresponding to a Cu-S bond, further supports this interaction (Milekhin et al., 2015; MincevaSukarova et al., 1997; Tailor et al., 2018). Moreover, the peaks typical of 1,10-phenanthroline ligand in the 100–1600 cm^− 1^ range (specifically, 408, 709, 1034, 1293, 1404 and 1586 cm^− 1^) exhibited a shift to higher wavenumbers (Zawada & Bukowska, 2000). These spectral changes reflect the incorporation of the ligand within the complex and are consistent with the formation of a Cu-CysPhe complex.

Electron paramagnetic resonance (EPR) experiments were conducted to verify coordination of the complex to hCtr1. EPR spectroscopy can report on direct atom coordination (nitrogen, oxygen, and sulfur) to the paramagnetic Cu(II) ion by evaluating the g and hyperfine values of the Cu(II) EPR spectrum **(Table ****S1**,** SI).** EPR measurements of the compound in the absence or presence of the full hCtr1 protein were performed. EPR data of the complex alone indicated Cu(II) as being coordinated by two nitrogen atoms from the Phe ligand and two sulfur atoms from the peptide (2N2S). In the presence of hCtr1, there were slight changes in the magnetic parameters, although coordination was still 2N2S, which suggested binding to the hCtr1 extracellular domain. This coordination differs from the coordination of Cu(II) to hCtr1, which presents a 2N2O or a 3N1O coordination. The coordination of Cu(II)-Phe without the peptide **(Figure S3**,** SI)** or hCtr1 also indicated 2N2O/3N1O-based coordination (two nitrogen and two oxygen atoms). However, in the presence of hCtr1, the EPR spectrum revealed some aggregation of Cu(II)-Phe without specific binding to hCtr1. The EPR data thus supported formation of a complex involving both the peptide with the ligand, and interaction withthe hCtr1 protein.

To further verify that the compound holds high affinity to the extracellular domain of hCtr1, isothermal titration calorimetry (ITC) studies were conducted on the N-terminal fragment (i.e., the first 52 amino acids) of the extracellular domain (**Figure S4**,** SI**). These experiments found that the binding affinity of the compound to the extracellular hCtr1 domain was 2.2 ± 0.4 nM. This is only slightly lower than the affinity of Cu(II) ions to hCtr1 (0.5 ± 0.2 nM).

In-vitro cell experiments were performed using non-radioactive Cu-CysPhe and human breast cancer cells (MCF-7), or mouse lymphoma cells (DA-3). Following incubation with Cu-CysPhe, the Cu(I) signal was evaluated using a bicinchoninic acid (BCA) assay (Brenner & Harris, 1995) (**Figure S5**,** SI**). BCA exhibits high affinity towards Cu(I) with a distinct absorption at 562 nm. An increase in intracellular Cu(I) levels was only observed under hypoxic conditions, confirming that the designed complex selectively targets hypoxic cells. Furthermore, the addition of Ag(I) ions, to inhibit Cu(I) transport via hCtr1 (Shenberger et al., 2015), did not result in an increase in the Cu(I)-BCA signal. This result is consistent with hCtr1-associated uptake pathways. Moreover, since Ag(I) ions coordinate with Met motifs in the N-terminal domain of hCtr1(Shenberger et al., 2015), these results support the hypothesis that Cu-CysPhe interacts with Met residues in the N-terminal domain of hCtr1.

The membrane permeability of the Cu-CysPhe complex was assessed using all-atom well-tempered metadynamics simulations with a POPC membrane model (Fig. 1c, **Figures S6 and S7**,** SI**). No membrane crossing events were observed. The simulations indicate that the complex encounters a prohibitively large free energy barrier, making transmembrane passage highly unlikely to occur. The same was observed for partially dissociated Cu-CysPhe complexes, regardless of the copper ion oxidation state (**Figure S6**,** SI**). These findings suggest that Cu-CysPhe cannot passively cross the membrane. In contrast, Cu-ATSM, known for its high membrane permeability (Colombie et al., 2015)^,^ (Vavere & Lewis, 2007; Xie et al., 2016), successfully traversed the lipid bilayer in our simulations, with an estimated free energy barrier of 30 kJ/mol (**Figure S7**, **SI**).

### Cell Experiments with Radioactive ^64^Cu-CysPhe

The ^64^Cu-CysPhe was prepared by binding the ^64^Cu(II) isotope to the Cys peptide and Phe ligand. ^64^CuCl_2_ was dissolved in PBS with Cys peptide and Phe at a 16:1.5:1 ratio. The molar activity of the radioactive complex was 4.28 ± 0.01 GBq/µmol (**Figures S8 and S9**,** SI**). HPLC data indicated that at least 95% of ^64^Cu ions were successfully bound to the peptide and ligand in buffer and that the complex was stable in Fetal bovine serum for at least 48 h (**Figure S10**,** SI**).

**Fig. 2:**
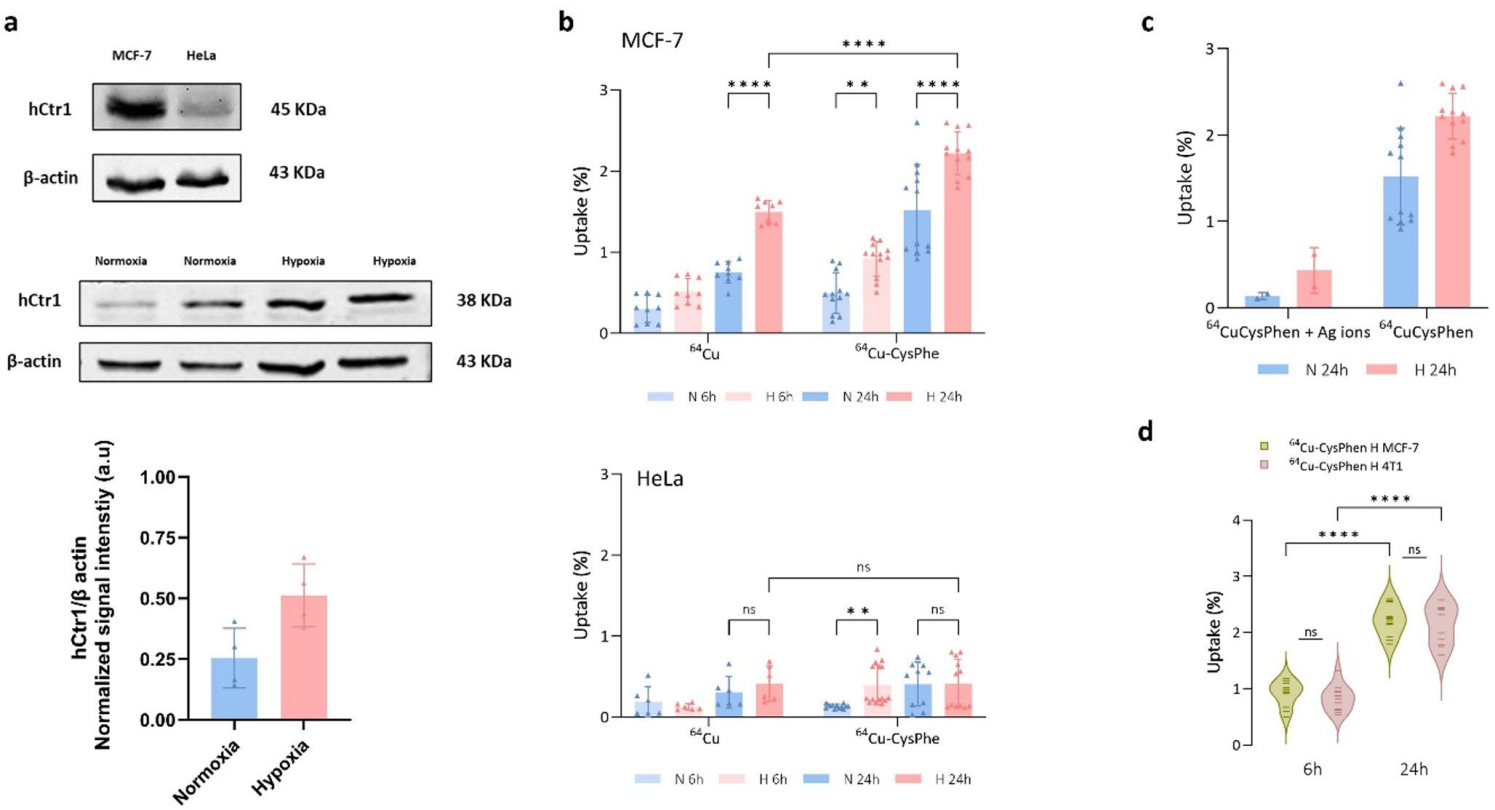
In-vitro cell experiments with ^64^Cu-CysPhe. **a**. Representative Western blot analysis of protein expression of hCtr1 and β-actin (internal control), in MCF-7 and Hela cells and in MCF-7 under hypoxic (H) and normoxic (N) conditions after 24 h, using antibodies directed against each protein. **b.** Uptake of ^64^Cu-CysPhe and ^64^Cu-Cl_2_ (termed ^64^Cu) (0.185 MBq) by MCF-7 and HeLa cell lines under H and N conditions after 6 and 24 h after compound application. **c**. MCF-7 cells challenged with radioactive ^64^Cu-CysPhe in the presence of Ag(I) ions after 24 h. **d**. Uptake comparison of ^64^Cu-CysPhe by MCF-7 human breast cancer cells and 4T1 mouse breast cancer cell. ****p < 0.0001

The ^64^Cu-CysPhe complex was subsequently added to cultures of breast or cervical cancer cells. (Figure 2a and b**).** Western blot experiments using antibodies to hCtr1 showed higher expression rate of hCtr1 in MCF-7 cells, as compared with HeLa cells. This expression was, moreover, elevated under hypoxic (H) versus normoxic (N) conditions (Fig. 2a, **Figures S11**,** SI**). For hypoxic conditions, cells were grown at 37 °C in an atmosphere containing 7–15% of CO_2_ and 0.1% O_2_ using hypoxia bag, and the hypoxic conditions were verified using Oxoid resazurin anaerobic indicator test. Figure 2b presents ^64^Cu-Cl_2_ and ^64^Cu-CysPhe (0.185 Mbq) uptake 6 and 24 h following compound administration to cells grown under normoxic (N) or hypoxic (H) conditions. In MCF-7 cells, a slight difference in complex uptake was observed between cells cultured under hypoxic vs. normoxic conditions 6 h post-administration (*p* = 0.0017). However, at 24 h, ^64^Cu-CysPhe uptake was significantly higher under hypoxic conditions compared to under normoxic conditions (*p* < 0.0001). This result indicates that the compound is retained longer in hypoxic cells. Furthermore, a comparison made with ^64^Cu-Cl_2_ showed that ^64^Cu-CysPhe exhibited greater cellular uptake.

In contrast with MCF-7 cells, hCtr1 expression in HeLa cells was very low. The reduced accumulation of ^64^Cu in HeLa cells, relative to MCF-7 cells, supports the important role of hCtr1 in ^64^Cu-CysPhe internalization. These experiments were repeated in the presence of Ag(I) ions, which block the Met motifs in the hCtr1’s N-terminal domain, as expected ^64^Cu-CysPhe uptake was reduced (Fig. 2c, **Figure S12**,** SI**). The results confirm that under both hypoxic and normoxic conditions, ^64^CuCysPhe uptake was negligible in the presence of Ag(I) ions. The correlation between compound uptake and hCtr1 expression, together with the observed interaction between Cu-CysPhe and hCtr1, suggests that uptake of Cu-CysPhe is dependent on hCtr1. Similar experiments were also conducted in 4T1 mouse breast cancer cells showed no significant differences in ^64^CuCysPhe uptake between human and murine breast cancer cells (Fig. 2d).

**Fig. 3:**
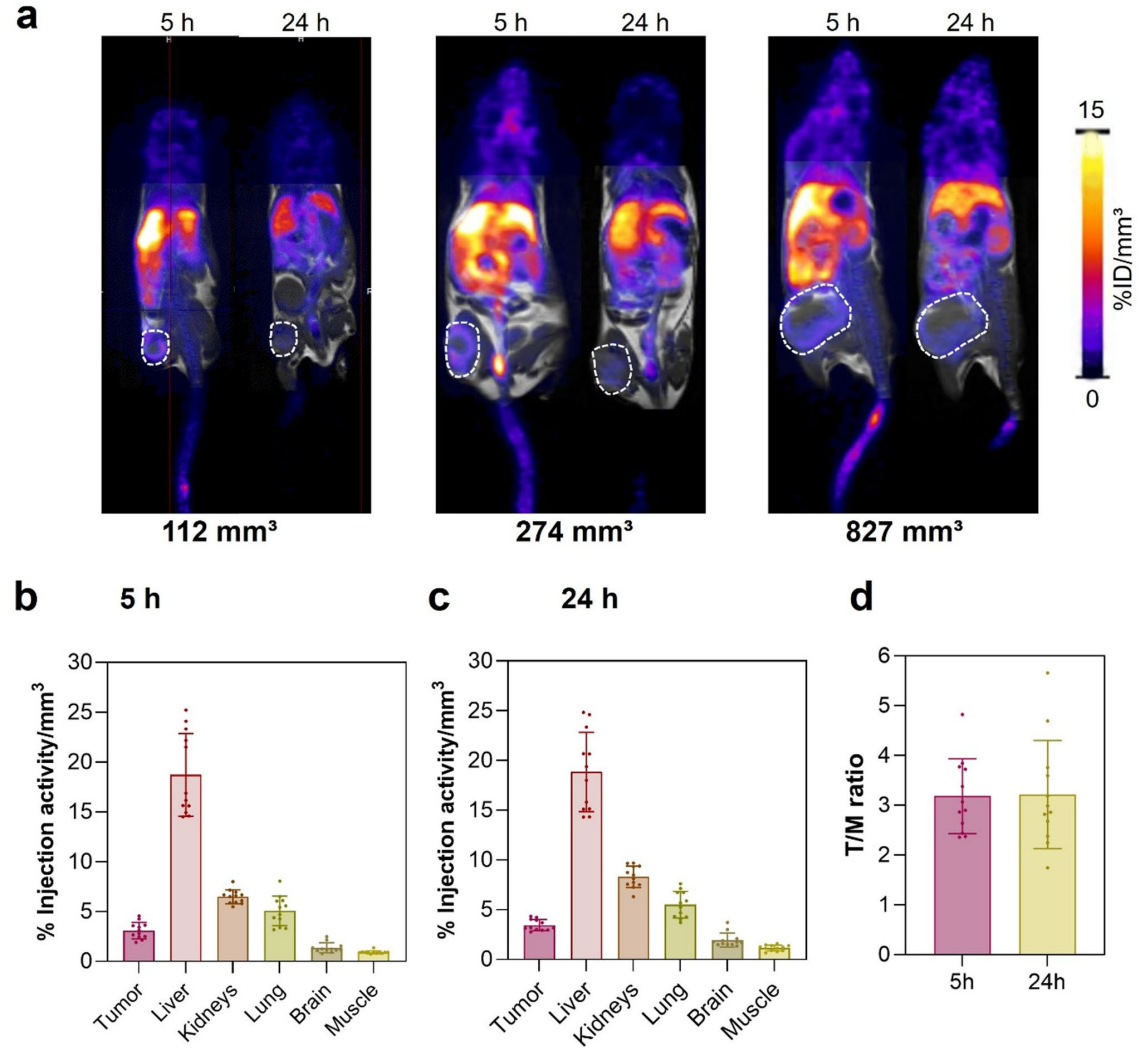
In-vivo experiments with ^64^Cu-CysPhe. **a.** Representative micro-PET-MRI images of BALB/c OlaHsd female mice with 4T1 cells tumors of increasing sizes acquired 10 days after sub-cutaneous implementation of 4T1 tumor cell into the lower back. ^64^Cu-CysPhe (8.1 ± 1MBq) was intravenously injected at time zero, and images were acquired at 5 h and 24 h after radiotracer injection (left and right respectively). The white circles mark the tumor, according to MRI. Tumor volume and imaging time after radiotracer injection are listed below and above each image, respectively. PET-MRI image quantitation of the biodistribution of ^64^Cu-CysPhe in the different organs at (**b**) 5 h post-injection; or (**c**) 24 h post-injection. **d**. The ratio between ^64^Cu-CysPhe uptake by the tumor and muscle 5 and 24 h post-injection. *N* = 12 mice

### In-vivo PET-MRI Imaging of ^64^Cu-CysPhe

Micro-PET-MRI imaging was performed on tumor-bearing mice 7–10 days following subcutaneous injection of murine 4T1 breast cancer cells into the lower back. The mice received an intravenous injection of ^64^Cu-CysPhe (8.1 ± 1 MBq), and tissue uptake was assessed at early (5 h) and late (24 h) time points post-administration (Fig. 3a-c, **Figure S13**,** SI**). Notably, tumors were clearly visualized in all mice, with pronounced delineation of tumor boundaries and necrotic regions. Uptake of ^64^Cu-CysPhe by smaller tumors was found to be higher, suggesting that necrotic regions lack viable cells. The imaging data revealed substantial accumulation of copper in the liver, intestines, and kidneys, consistent with the biodistribution profile of copper-based compounds (Anderson et al., 2001; Lewis et al., 1999; Qi et al., 2019). Importantly, low accumulation of the compound was observed in the lungs and brain. This observation further illustrates the elevated uptake of ^64^Cu-CysPhe in tumors, as compared to muscle tissue, yielding a tumor-to-muscle (T/M) ratio of 3.1 ± 0.5 at 5 h and 3.25 ± 1.4 at 24 h (Fig. 3d).

**Fig. 4:**
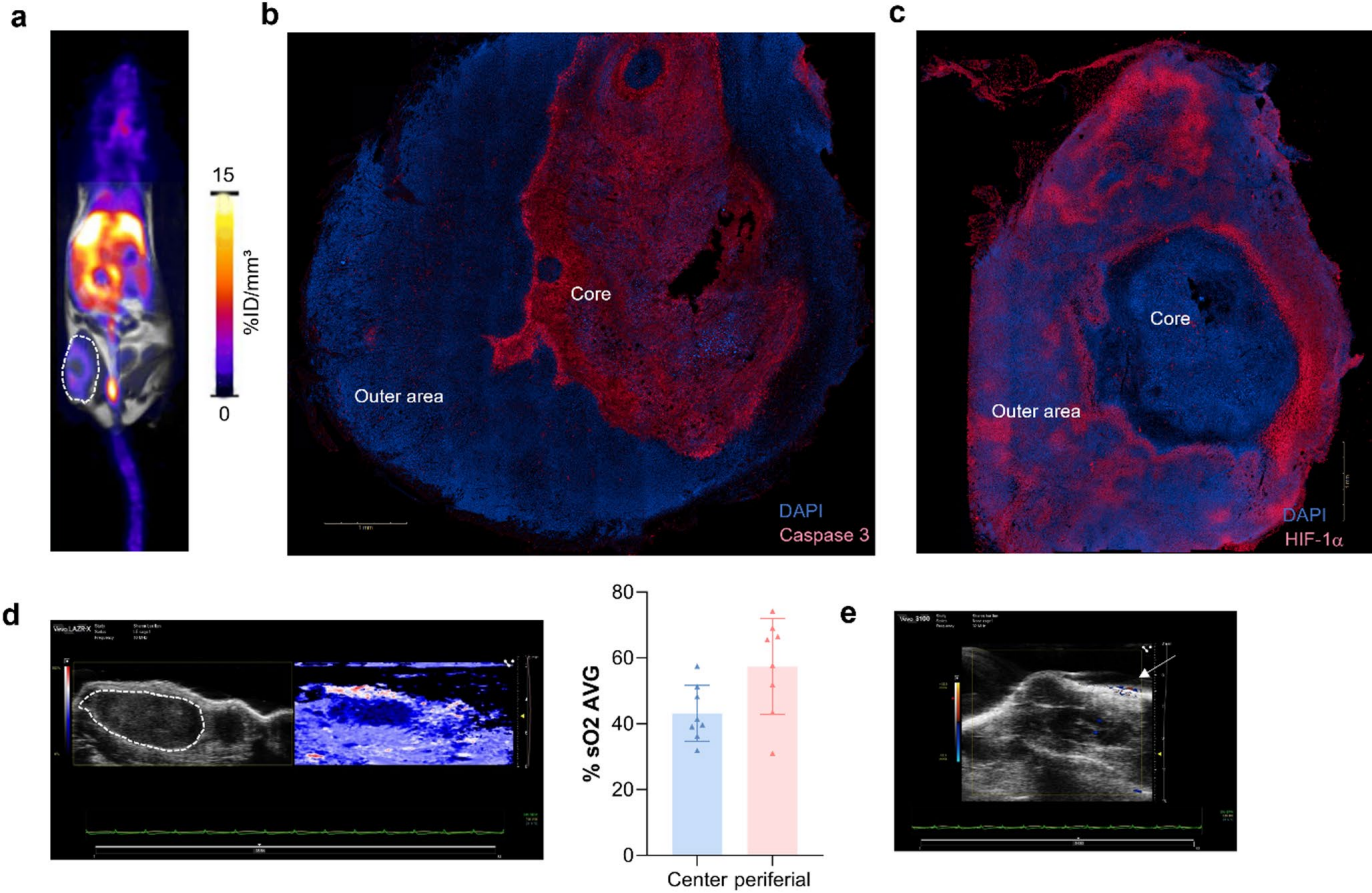
^64^Cu-CysPhe accumulation in hypoxic regions. **a.** Representative PET-MRI image of BALB/c OlaHsd female mice with 4T1 breast tumor acquired 5 h after intravenous injection of ^64^Cu-CysPhe (8.473 MBq). The white circle marks the tumor border. **b.** Cell apoptosis at the center of this tumor, as demonstrated by caspase 3 immunostaining. **c.** Tissue hypoxia at the breast cancer tumor periphery, as demonstrated by HIF-1α immunostaining. **d**. Photoacoustic ultrasound and sO_2_ levels in central and peripheral tumor regions. **e.** Doppler ultrasound showing that tumor vasculature is mainly located at the tumor rim

To correlate the PET-MRI findings with actual tumor cellularity and hypoxic status, immunohistochemical (IHC) staining was performed. Caspase-3 serves as a marker for apoptosis, while HIF-1α indicates tissue hypoxia. Figure 4a presents a representative PET-MRI scan obtained 5 h post ^64^Cu-CysPhe injection, showing tracer accumulation at the tumor margins, with an absence of signal in the tumor core. IHC staining confirmed that the outer tumor region, which corresponded to the PET-MRI signal, was hypoxic (HIF-1α-positive staining, (Fig. 4c)). At the same time, the central tumor region exhibited extensive apoptosis, as indicated by caspase 3-positive staining (Fig. 4b). These findings suggest that ^64^Cu-CysPhe does not penetrate the necrotic core yet effectively delineates tumor boundaries (results from additional tumors are provided in **Figure S14**,** SI**).

Another critical factor influencing radiotracer distribution is tumor vascularization. To assess vascular status, tumor-bearing mice underwent photoacoustic and Doppler ultrasound imaging two days before PET-MRI scanning. Photoacoustic ultrasound measurements indicated significantly higher oxygenation levels at the tumor periphery (Fig. 4d). Furthermore, Tumor vasculature was predominantly localized to the tumor rim (Fig. 4e).

### Comparing ^64^Cu-CysPhe to Other Tracers

**Fig. 5:**
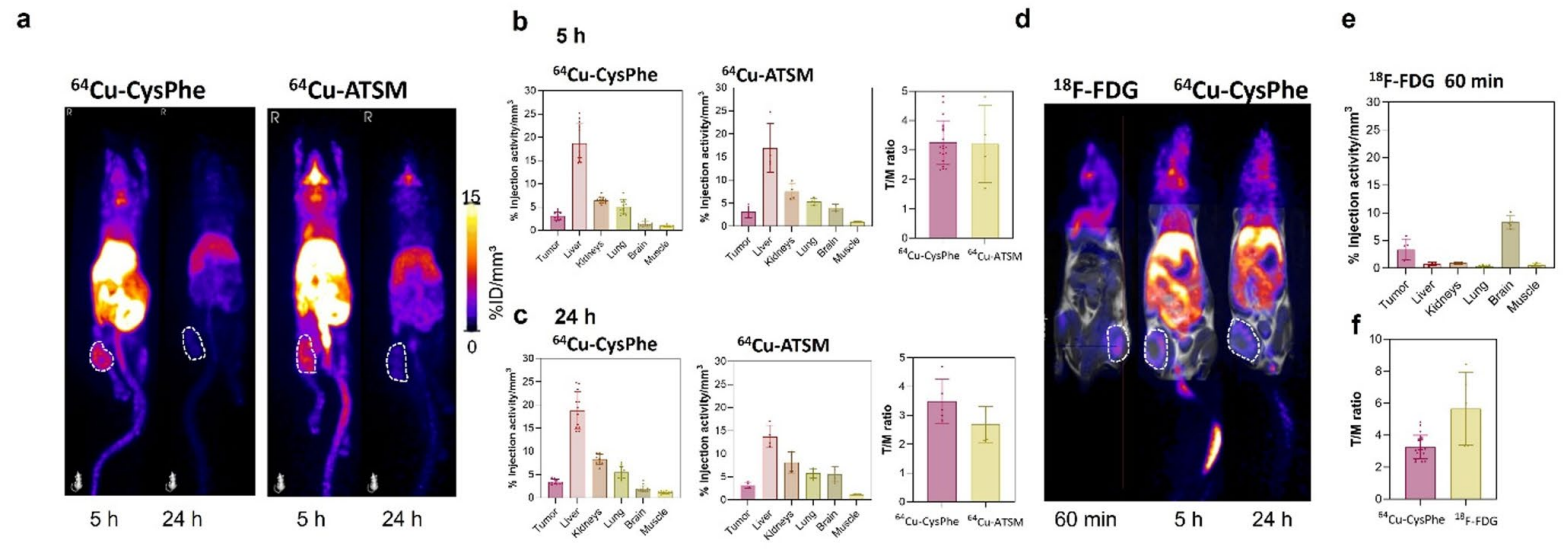
A comparison between ^64^Cu-CysPhe and ^64^Cu-ATSM, ^18^F-FDG distribution. **a**. PET-MRI images of BALB/c OlaHsd female mice with 4T1 breast tumors sized 220 ± 5 mm^3^ were acquired at 5 and 24 h after intravenous injection ^64^Cu-CysPhe (left) or ^64^Cu-ATSM (right). The white circles mark the tumor borders. Biodistribution of ^64^Cu-CysPhe and ^64^Cu-ATSM at (**b**) 5 h and (**c**) 24 h post-injection. *N* = 5 mice at 5 h and 24 h with (^64^Cu-CysPhe); *N* = 4 with (^64^Cu-ATSM). **d.** PET-MRI images of representative BALB/c OlaHsd female mouse with ^18^F-FDG (10.62 MBq) was intravenously injected, and mice were scanned for 20 min starting 40 min post-injection. ^64^Cu-CysPhe were injected 24 h later into the same mice. The white hatched line marks the tumor. Tumor sizes detected by MRI during the ^18^F-FDG experiment were 283 mm^3^, and 363 mm^3^ after 24 h. **e**. Biodistribution of ^18^F-FDG. **f**. Tumor to muscle ratio for ^64^Cu-CysPhe (5 h) and ^18^F-FDG. *N* = 5 mice at 5 h and *N* = 5 for (^18^F-FDG)

We assessed the efficacy of ^64^Cu-CysPhe for tumor detection relative to other PET tracers, specifically ^64^Cu-ATSM (Fig. 5, **Figure S15**,** SI**) and ^18^F-FDG (Fig. 5, **Figure S16**,** SI**). Figure 5a presents representative PET-MRI images of mice with similar tumors size (220 ± 5 mm^3^), imaged with either ^64^CuCysPhe (left) or ^64^Cu-ATSM (right) radiotracers. The biodistribution obtained from the PET-MRI image quantitation of both tracers was evaluated at 5 h (Fig. 5b) and 24 h post-injection (Fig. 5c). No statistically significant differences were observed in T/M ratios between the tracers 5 h post-injection (3.06 ± 1.3 for ^64^Cu-ATSM and 3.1 ± 0.5 for ^64^Cu-CysPhe). In-vitro cell studies revealed that ^64^Cu-ATSM exhibits greater accumulation in 4T1 cells compared to ^64^Cu-CysPhe (**Figure S17**,** SI**), which is consistent with computational modelling suggesting ^64^Cu-ATSM presents higher membrane permeability than ^64^Cu-CysPhe. In-vivo imaging and biodistribution analyses after 24 h, revealed a T/M ratio of 2.47 ± 0.3 for ^64^Cu-ATSM and 3.25 ± 1.4 for ^64^Cu-CysPhe. Moreover, PET-MRI biodistribution analysis indicated a two-fold increase of ^64^Cu-ATSM accumulation in the brain, as compared to ^64^Cu-CysPhe.

Next, the distribution of ^64^Cu-CysPhe was compared with that of ^18^F-FDG (Fig. 5, **Figure S16**,** SI**). Mice were scanned with both tracers, for 20 min starting 40 min post ^18^F-FDG injection, or 5 h and 24 h following ^64^Cu-CysPhe injection. Figure 5d presents PET-MRI images of mice administered both ^18^F-FDG. PET-MRI biodistribution analysis revealed lower ^18^F-FDG accumulation in the liver, kidneys and lungs but higher uptake in the brain (Fig. 5e). Moreover, the experiments showed a higher T/M ratio for ^18^F-FDG than for ^64^Cu-CysPhe (5.66 ± 2.27 vs. 3.1 ± 0.5) (Fig. 5f).

## Discussion

Given the clinical importance of identifying and characterizing hypoxic tumors, together with the growing need for personalized therapeutic strategies, the development of novel diagnostic radiotracers remains highly significant. In this study, we developed a ^64^Cu-based radiotracer inspired by the biological mechanisms of cellular copper transport involving the main human copper transporter, hCtr1. The designed radiotracer targets solid tumors through copper metabolism related pathways, while its enhanced uptake and prolonged retention in hypoxic tumor regions reflect both tumor presence and the associated hypoxic microenvironment.

^64^Cu-CysPhe is characterized by a cellular uptake mechanism that differs from passive membrane diffusion. The dependence of tracer uptake on hCtr1-associated processes was supported by several complementary in-vitro experiments and computational analyses. ITC measurements demonstrated interaction between the complex and the extracellular domain of hCtr1, while cellular uptake studies showed correlation between tracer accumulation and hCtr1 expression under different experimental conditions. In addition, all-atom molecular dynamics simulations indicated that Cu-CysPhe is unable to cross lipid membranes through passive diffusion, therefore its uptake is more likely involve active or facilitated transport mechanisms associated with copper transport pathways. Furthermore, both in-vitro and cell-based assays showed selectivity and enhanced retention time of the radiotracer under hypoxic conditions. While the present study does not demonstrate direct transport of the intact complex through hCtr1 under physiological conditions, the collective experimental observations suggest involvement of hCtr1. Additional mechanistic studies will be required to fully distinguish between intact complex transport and alternative uptake and transfer processes.

This study was conducted as a preliminary investigation aimed at assessing the feasibility of the procedures and to obtain an initial estimate of the effect size. The ^64^Cu-CysPhe compound was evaluated in a series of PET-MRI imaging studies performed on mice with breast cancer tumors. The in-vivo data indicated T/M ratios exceeding 3.0 for ^64^Cu-CysPhe in tumors ranging from 70 to 1500 mm³, in images acquired 5 and 24 h post-injection. Tumors boundaries were clearly delineated, suggesting rapid clearance from normoxic tissues. Correlation between the PET-MRI images and the IHC data indicated that the complex does not penetrate necrotic tumor cores but can report on hypoxic regions. Moreover, ultrasound imaging confirmed the absence of vascularization within the tumors.

Comparison experiments, although limited by the small sample size, showed that ^64^Cu-CysPhe holds similar tumor uptake as ^64^Cu-ATSM and lower accumulation in the brain (Blockhuys et al., 2021; Ge et al., 2022; Qasem et al., 2022; Zhang et al., 2022, 2023). Comparison with ^18^F-FDG showed that the T/M ratio of ^64^Cu-CysPhe was lower, albeit the timepoints compared were vastly different. While ^18^F-FDG remains an excellent and affordable radiotracer for tumor detection, it suffers from low specificity, since glucose uptake is not exclusive to malignant cells. Therefore, our goal was to propose a radiotracer in addition to ^18^F-FDG, which may provide complementary information on tumor state.

In summary, we developed the ^64^Cu-CysPhe complex based on the cellular copper cycle. This complex offers several advantages, including the 12.7 h half-life of ^64^Cu that provides a suitable time window for imaging following the reduction of ^64^Cu(II) to ^64^Cu(I) in a hypoxic environment. The synthesis of the compound is straightforward, achieving over 95% binding efficiency, as validated by HPLC UV/Gamma detection. Additionally, ^64^Cu-CysPhe exhibits good uptake in hypoxic tumors, and rapid clearance from normoxic tissues. Thus, it represents a novel radiotracer for diagnosing hypoxic conditions in tumors.

Finally, since hypoxia is not exclusive to solid tumors but is also associated with ischemia and neurodegenerative pathologies such as α-synuclein plaques and Aβ amyloids, this newly developed radiotracer has the potential for use in the diagnosis of stroke, Alzheimer’s disease, and Parkinson’s disease (Park et al., 2015; Raz et al., 2015; Syme et al., 2004) .

## Supplementary Information

Below is the link to the electronic supplementary material.

Supplementary Material 1

## Data Availability

No datasets were generated or analysed during the current study.
