# Supplementary material for: 64Cu Hypoxia Imaging Radiotracer Targeting the Human Copper Transporter
Source: Neuromolecular Med. 2026 Jun 29;28(1):40. doi: 10.1007/s12017-026-08939-4 (PMC13315494; doi:10.1007/s12017-026-08939-4)
Supplement: Supplementary file 1 — Supplementary Material 1 [file 12017_2026_8939_MOESM1_ESM.pdf]

Supporting Information

^{64}Cu Hypoxia Imaging Radiotracer Targeting the Human Copper Transporter

Shelly Meron[a], Yulia Shenberger[a], Ravit Madar[b, c, d], Jana Aupic[e], Nathalie Abudi[f, g], Fabio Lapenta[h], Melanie Hirsch[a], Odelia Orbaum Harel[b], Lukas Hofmann[a], Alessandra Magistrato[e], Eitan Okun[b, c, d], Rinat Abramovitch[f, g], and Sharon Ruthstein[a, *].

[a] Department of Chemistry, Institute of Nanotechnology and Advanced Materials, Faculty of Exact Sciences, Bar-Ilan University, Ramat-Gan 5290002, Israel

[b] The Mina and Everard Goodman Faculty of Life Sciences, Bar-Ilan University, Ramat Gan 5290002, Israel

[c] The Gonda Multidisciplinary Brain Research Center, Bar-Ilan University, Ramat Gan 5290002, Israel

[d] The Paul Feder Laboratory for Alzheimer's disease research, Bar-Ilan University, Ramat Gan 5290002, Israel.

[e] CNR-IOM at SISSA, Trieste 34135, Italy

[f] Wohl Institute for Translational Medicine, Hadassah Medical Center, Jerusalem 91120, Israel

[g] Faculty of Medicine, Hebrew University of Jerusalem, Jerusalem 91904, Israel

[h] Laboratory for Environmental and Life Sciences, University of Nova Gorica, Nova Gorica 5000, Slovenia

Table of Contents

Materials and Methods **3**

The human copper transporter (hCtr1) expression and purification 3

Extracellular hCtr1 N-terminal domain expression and purification 3

Solid-phase peptide synthesis 3

HRMS analysis 4

^{64}Cu-CysPhe preparation 5

Cu-CysPhe preparation 5

Cell experiments 5

Cell experiments with radioactive ^{64}Cu-CysPhe 6

HPLC 6

^{64}Cu-ATSM preparation 6

Electron Paramagnetic Resonance analysis 6

Western Blot 7

Raman Measurements 8

Isothermal titration calorimetry (ITC) 8

Computational Methods **8**

System set-up 8

Metadynamics 9

Statistic **9**

***In-vivo* experiments** **9**

In-vivo micro PET-MRI scanning 9

Ultrasound (US) and photoacoustic (PA) imaging 10

Immunohistochemistry (IHC) 10

ITC measurements **11**

Data from cell experiments with non-radioactive Cu-CysPhe **11**

Computational measurements **12**

Molar activity **14**

Unprocessed Images of Western blots and corresponding gels **16**

Data from cell experiments using radioactive ^{64}Cu-CysPhe in the presence of Ag(I) ions **16**

Imaging PET-MRI data **17**

Data from 4T1 cell experiments using radioactive ^{64}Cu-CysPhe Vs ^{64}Cu-ATSM **19**

References **20**

Materials and Methods

The human copper transporter (hCtr1) expression and purification

hCtr1 expression was carried out as previously described (Walke et al., 2022). hCtr1-containing constructs were prepared by PCR amplification and ligated into a modified pFastBac (pK503-9) insect cell expression vector, encoding an N-terminal FLAG tag. To produce baculovirus for hCtr1 expression, recombinant bacmid was extracted and transfected into Sf9 cells using Cellfectin II Reagent (ThermoFisher) according to procedures described in the Bac-to-Bac instruction manual (Invitrogen). Insect Sf9 cells were grown at 27 °C in protein-free ESF 921 insect cell culture media (Expression Systems) in roller-bottles and incubated for three days post-infection. The cells were harvested and resuspended into buffer solution (400 mM NaCl 10% glycerol, 20 mM HEPES buffer, pH = 7.4), lysed and centrifuged at 40 rpm for 40 min. The pellet was resuspended with buffer solution (1.5% Triton X-100, 200 mM NaCl, 10% glycerol, 20 mM HEPES buffer pH = 7.4), and incubated over night at 4°C. The suspension was centrifuged again at 40 rpm for 40 min. 3 mM of $CaCl_2$ were added to the supernatant, which was loaded onto an anti-FLAG M1 agarose affinity gel (Sigma) column pre-equilibrated with TBS buffer (150 mM NaCl, 50 mM Tris-HCl pH=7.4) and incubated over night at 4°C. The column was washed with TBS buffer and after applying a 5 mM EDTA elution buffer protein-containing fractions were collected and analyzed by sodium dodecylsulfate polyacrylamide gel electrophoresis (SDS-PAGE) (14% glycine) and silver staining (Walke et al., 2022).

Extracellular hCtr1 N-terminal domain expression and purification

DNA coding for amino acids 1 to 52 of hCtr1 protein (NCBI Reference Sequence NP_001850.1) was codon optimized for *E. coli*, ordered from Twist Bioscience and inserted via Gibson assembly (New England Biolabs) into the pET41a+ (Novagen) expression vector. *E. coli* LOBSTR-BL21(DE3) competent cells (Fisher Scientific) were transformed by heat shock with the construct and selected on kanamycin (50 µg/mL) supplemented LB plates. Pre-cultures were grown with agitation at 37°C overnight, then diluted to OD_{600} 0.1 in 2 L of LB media supplemented with kanamycin, grown until OD_{600} 0.6 and induced to express the protein upon induction with 1 mM of isopropyl β-d-1-thiogalactopyranoside (IPTG) for 15 h at 25°C. The harvested cellular pellet was frozen and thawed two times and then resuspended in 30 mL of buffer A (50 mM Tris-HCl pH 8.0, 150 mM NaCl), supplemented with EDTA-free Protease Inhibitor Cocktail (Roche) and DNase I (Thermo Fisher scientific) in ratios specified by the manufacturers. Cells were sonicated and the lysate was clarified by centrifugation at 16.000 RCF for 20 min at 4°C. The soluble fraction was applied to a 5 mL HisTrap column (Cytiva), which was then washed with buffer A supplemented with 40 mM imidazole and finally eluted in buffer A containing 250 mM imidazole.

Solid-phase peptide synthesis

A basic peptide synthesis strategy was used with Fmoc (9-fluorenylmethoxy-car-bonyl)-protected amino acids and a resin linkage that are acid-labile. To a 12 ml syringe, 300 mg of resin (GL Biochem (Shanghai)) and 5 ml of DMF (dimethylformamide, Bio-Lab) were added and incubated for 20 min. The solution was removed from the syringe and 5 ml of piperidine solution (66.66% piperidine and 33.33% DMF) was added for 15 min to remove the protecting group. Subsequently, the syringe was rinsed five times with 5 ml of DMF. An amino acid (400 mg) (GL Biochem (Shanghai) or Bio-Lab), 275 mg HBTU (Hexafluorophosphate Benzotriazole Tetramethyl Uronium, Sigma-Aldrich), 224 µl DIPEA (N,N,-diisopropylethylamine, Bio-Lab), and 5 ml DMF were added to a vial and mixed until a uniform and clear solution was obtained. Then, the vial contents were transferred to the syringe and shaken for 90 min. Then, the syringe was rinsed three times with 5 ml DMF and a Kaiser test was performed. After the Kaiser test, the protecting group was removed with 5 ml of piperidine solution for 15 min and the

process was repeated. At the end of the process, the syringe contents were dried with 5 ml DCM (Bio-Lab) and cleavage was performed to obtain the peptide.

Cleavage: A mixture of 5.4 ml TFA (Bio-Lab), 300 µl H_2O, 150 µl thioanisole (Sigma-Aldrich), 150 µl triisopropylsilane (Sigma-Aldrich), and 300 µl EDT (Sigma-Aldrich) were added to a vial and transferred to the syringe with subsequent shaking over two hours. The contents of the syringe were then transferred into a vial, 65 µl of bromotrimethylsilane (Sigma-Aldrich) were added and the mixture was transferred into the syringe. The syringe was then shaken for 30 min. The contents were then transferred to a 50 ml test tube and cold ethyl ether (Bio-Lab) was added. The tube was closed with parafilm and vortexed. Subsequently, the test tube was centrifuged for 5 min at 4000 RPM and 8°C. Finally, the top liquid was removed, and the process was repeated four times. The collected samples were dried under nitrogen and analyzed by mass spectrometry (MS).

HRMS analysis

Complex was analyzed using an Agilent Technologies system consisting of a 6545 QTOF mass spectrometer equipped with an electrospray ionization (ESI) interface. Acetonitrile and H2O were used as the mobile phase throughout the run. ESI was operated in a positive mode. MS parameters were optimized using the fragmentor voltage 180 V, source temperature 300 °C, drying gas flow 8 L/min, nebulizer pressure 40 psi, sheath gas 12 L/min at 400 °C. In the obtained ESI-MS spectrum, intense peaks corresponding to the free peptide, phenanthroline ligand, and phenanthroline ligand-Cu63 were observed, reflecting their high ionization efficiency under positive ESI conditions. Such behavior is commonly reported for small aromatic chelators and protonated peptides due to their favorable gas phase basicity and charge delocalization (Gianelli et al., 2001; Maccarrone et al., 2009; Plonka et al., 2021).

Importantly, additional peaks matching the calculated isotopic pattern of the peptide Cu phenanthroline complex were detected, confirming successful complex formation.

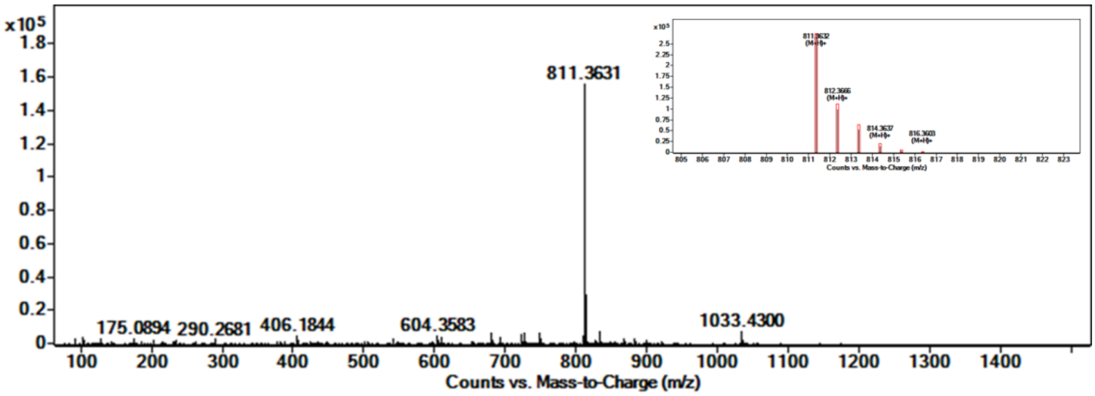

Figure S1: HRMS spectrum of Cys peptide sequence KSMAACAM. The inset highlights the excellent match between the experimental isotopic distribution and the calculated pattern for peptide.

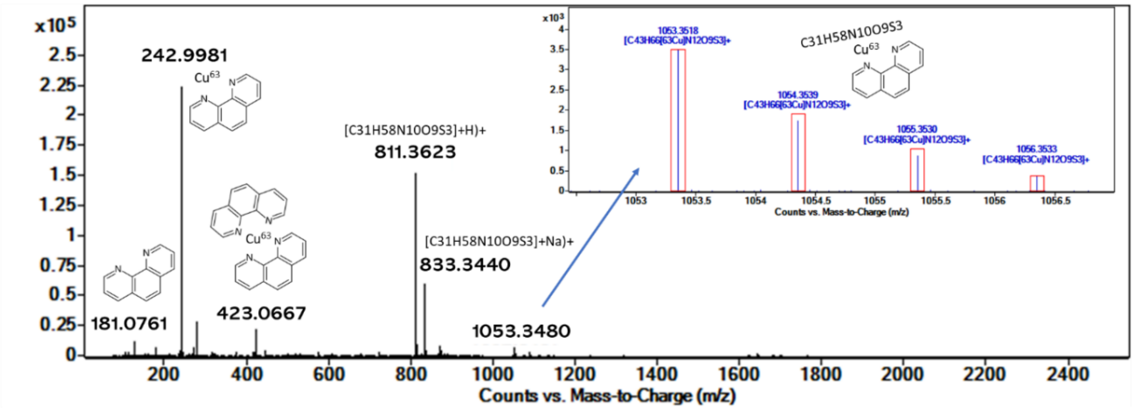

Figure S2: HRMS spectrum of Cu-CysPhe complex. The inset highlights the excellent match between the experimental isotopic distribution and the calculated pattern.

^{64}Cu-CysPhe preparation

Radioactive ^{64}Cu from A.C.O.M (Advanced Center Oncology Macerata – S.R.L) Italy was shipped to Bar-Ilan University, Israel. ^{64}Cu in 0.1 N HCl, pH=1 was diluted to 4-5 ml with acetate buffer, pH=4-5. Preparation of the radioactive complex was as follows: First the pH of the ^{64}Cu was adjusted to pH=6.9-7.2 with 10 M NaOH (Bio-Lab) and PBS (Sartorius Biological industries). ^{64}Cu, 24 mM KSMAACAM (Cys) peptide and 10 mM 1,10 Phenanthroline (Phe, Sigma-Aldrich) at a 16:1.5:1 ratio, were dissolved in PBS, mixed, and heated for least 30 min in 37°C bath. Complex formation was verified by HPLC.

Cu-CysPhe preparation

At a ratio of 1:4:2 24 mM $CuCl_2$ (Tzamal D-chem Laboratories), 24 mM Cys peptide and 10 mM Phe were dissolved in DDW, mixed by vortex and stirred for least 30 min in a 37°C shaker incubator. The final Cu-CysPhe concentration was 2.155 mM.

Cell experiments

The present study utilized two tumor-derived cell lines, namely; (1) MCF-7 cells, an estrogen receptor (ER)-positive, progesterone receptor (PR)-positive, and human epidermal growth factor receptor 2 (HER2)-negative human breast cancer cell line; (2) Hela cells, a human Papillomavirus 18 (HPV18) positive human cervical adenocarcinoma cell line derived from cervical cancer; (3) DA-3 cells, a murine mammary adenocarcinoma cell line derived from BALB/c mice; and (4) 4T1 cells, a highly metastatic murine mammary carcinoma cell line derived from BALB/c mice that closely models stage IV human breast cancer. Cancer cells were seeded in T75 flasks at $1{\cdot}10^6$ cells/flask in high glucose Dulbecco's modified Eagle's medium (DMEM, Sigma Life Science) or Eagle's minimum essential medium (EMEM, Sigma Life Science) containing 10% fetal bovine serum (FBS, Gibco), together with 1% penicillin-streptomycin-neomycin solution (PSNS, Sartorius Biological industries) and 1% L-glutamine (Sartorius Biological Industries) and incubated at 37˚C in an atmosphere of 5% CO_2. The cells were passaged weekly by diluting four-fold. After incubation (80% confluency has been reached), dead cells and growth medium were removed, 5 ml trypsin solution (Sigma-Aldrich) were added to the flasks, which were returned to the incubator for 5 min. The trypsin was subsequently diluted with 5 ml media, followed by centrifugation at 300 rpm for 5 min at 21°C.

Two ml of the cells culture were added to the well of 6-well plates such that each well contained $2.5{\cdot}10^5$ cells. Two days later, when the cells reached 90% the required number ($1{\cdot}10^6$ cells), the medium was replaced with 2 ml fresh medium and Cu-CysPhe was added to a final concentration of 0.25 or 0.5 mM per well. To create hypoxic conditions, an anaerobic atmosphere generation bag was added to the box, which were then sealed with a paraffin. Under normoxic conditions, the cells were incubated for an entire day at 37°C and 5% CO_2, while under hypoxic conditions, the plates were kept

at 37°C in an atmosphere containing 7-15% of CO_2 and 0.1% O_2 using hypoxia bag (Oxoid™ AnaeroGen™ 2.5L Sachet). Hypoxic conditions were verified using Oxoid resazurin anaerobic indicator test (Thermo Scientific, Sigma-Aldrich). Aliquots were removed after 4 h, 6 h and 24 h, the medium and the cells were washed with 3 ml PBS. Cold RIPA buffer (300 µl, Sigma life science) was added to the wells for 10 min at 4°C. For quantification of the Cu(I) concentration, a Pierce BCA protein assay kit (Protein Biology Thermo Scientific) was used. A 300 µl volume solution of the BCA protein assay kit was added and heated to 42°C for 10 min. Afterwards, samples were measured at a wavelength of 562 nm in UV-VIS spectrophotometer.

Cell experiments with radioactive ^{64}Cu-CysPhe

Two ml of cell culture were added to each well of 6-well plate at a final cell count of $2.5{\cdot}10^5$. Once the cell count reached $1{\cdot}10^6$, the medium was replaced with 2 ml fresh medium and 150 µl, 0.185 MBq of the complex were added to each well. The cells were grown under hypoxic or normoxic conditions, generated as above. At selected time points, the medium was removed, three cells were washed with 3 ml PBS, 600 µl RIPA buffer and the cells were solubilized by scrapping. Then floating cells were measured for radioactivity using Wizard^2 1-detector gamma counter (PerkinElmer). All measurements were taken after fixing the detector efficiency to 100%. Ppercentages uptake normalized to cell number.

HPLC

A Shimadzu model LC-2050 series HPLC system (pressure resistance 50 MPa) comprise of a UV-Vis detector and gamma detector (Gabi Nova 1.4 Mid Energy Probe). A Phenomenex Kinetex 2.6 µm EVO C18 100A | LC column (100 x 2.1 mm) was used. Detection was performed at 280 and 220 nm for both organic materials (peptide and ligand Phe). The elution solvents were A (water with 0.1% TFA) and B (acetonitrile with 0.1% TFA, Bio-Lab). The samples were eluted according to the following gradient: 100% A/0% B to 0% A/100% B, at a flow rate of 0.2 ml/min and a run of 37 min. The run was performed at room temperature. The sample injection volume was between 0.05-0.1 ml. Identification of compounds was achieved by comparing running times of the organic materials to that of the radioactive isotope. Data was collected and processed using GINA chromatography software.

^{64}Cu-ATSM preparation

The ATSM ligand was synthesized as previously reported (Walke et al., 2021; Walke & Ruthstein, 2019). 4-methyl-3-thiosemicarbazide (1.2 g, 11.4 mmol) was dissolved in ethanol (50 ml) with constant heating and stirring. Then, an ethanolic solution of diacetyl (2,3_butanedione) (0.5 ml, 5.7 mmol) was added dropwise into the solution. Next, 5-6 drops of glacial acetic acid were added. The reaction mixture was refluxed at 60-70°C for 4 h. A white colored precipitate formed. The flask was kept at 4°C overnight for complete precipitation. The next day, a pale, yellow precipitate corresponding to ATSM was obtained and washed with ethanol and diethyl ether 3-4 times each. ^{64}Cu-ATSM was prepared by initially adding the ^{64}Cu stock solution (in 0.1 N HCl) to 4 ml of 200 mM glycine (Thermo Scientific) in aqueous solvent, pH=5-6. H_2ATSM in dimethyl sulfoxide (DMSO) (20:1 by volume) was added and the solution was mixed.

Electron Paramagnetic Resonance analysis

Continuous wave (CW)-EPR spectra were recorded using an E500 Elexsys Bruker spectrometer operating at 9.0−9.5 GHz and equipped with a high-sensitivity CW resonator. Spectra were recorded at low temperature (130 ± 5 K, 9.3 GHz) at a microwave power of 20.0 mW, modulation amplitude of 1.0 G, a time constant of 80 ms, and a receiver gain of 60.0 dB. The samples were measured in a 1.0-mm quartz tube, which was placed in a 4.0 mm quartz tube for cooling (Wilmad-LabGlass, Vineland, NJ). CW-EPR simulations were carried out using MATLAB, with the EasySpin toolbox (Stoll & Schweiger,

2006). The fitted parameters are listed in Table S1. The coordination environment was obtained from Peisach et al. (Peisach & Blumberg, 1974).

Sample	g-tensor	A-tensor [MHz]	% species	Line-width [G]	Coordination
**Cu(II)_Cys_Phe**	[2.08 2.12]	[20 470]	90	6.0	2N2S
	[2.07 2.25]	[20 480]	10	5.0	2N2O/3N1O
**Cu(II)_Cys_Phe_hCtr1**	[2.08 2.21]	[20 460]	85	9.0	2N2S
	[2.06 2.28]	[20 420]	15	2.8	2N2O/3N1O
**Cu(II)_hCtr1**	[2.06 2.28]	[20 470]	90	4.0	2N2O/3N1O
	[2.05 2.34]	[25 390]	10	3.0	4O
**Cu(II)_Phe**	[2.06 2.28]	[20 470]	80	3.0	2N2O/3N1O
	[2.045 2.36]	[25 390]	20	3.0	4O

Table S1: EPR parameters for Cu(II) bound to compound/hCtr1/ligand obtained from EasySpin simulations using Pepper program.

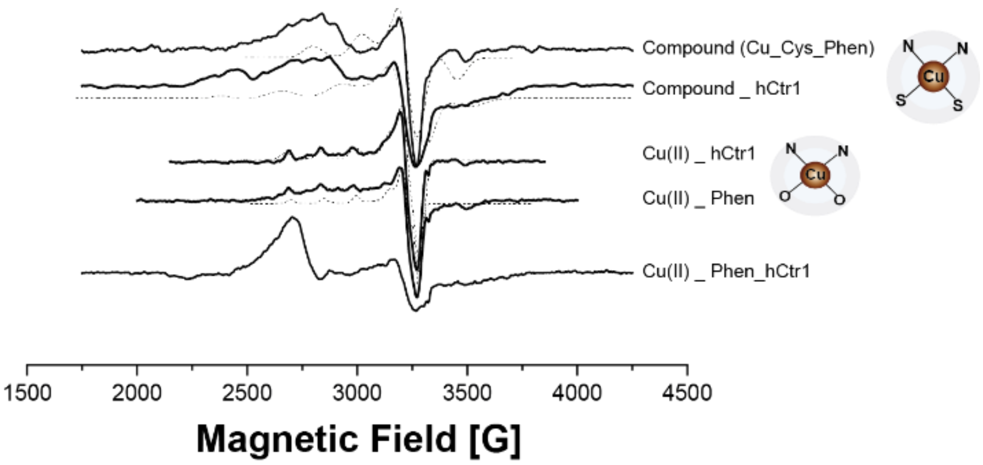

Figure S3: CW EPR spectra for Cu(II) bound to peptide and ligand (compound), ligand, and Cu(II)Cl$_2$, in the absence or presence of hCtr1. The suggested schematic coordination is shown on the right. [Cu(II)] = 140 µM in all experiments.

Western Blot

Tested cells were grown in cell culture flasks and incubated at 37°C with 5% CO_2 for a few days. After incubation, the culture was washed twice with 3 ml cold PBS. Afterwards, the cells were scraped, 2 ml of PBS was added, and the suspension was centrifuged for 5 minutes at 500 G and 4°C. The suspension was removed, PBS 500 µl was added and the suspension was transferred to an Eppendorf 1.5 ml and centrifuged for 5 minutes at 500 G, and 4°C. The suspension was removed, and RIPA Buffer was added with protease inhibitors (Coc 1:50 and PMSF 1:500) to lyse the cells. The lysed culture was then put on ice for 20 min**.** The samples were then centrifuged for 15 min at 11,000 rpm and 4°C and the resulting suspension was removed into a new Eppendorf. Sample concentration was determined as follows; preparation of a stock solution with protein assay dye reagent concentrate – Bradford in a ratio of 1:4. Into a 96 well plate was added; 100 µl from the stock, 99 µl DDW and 1 µl from the sample. An absorption test was performed on a synergy H1 device with a wavelength of 595 nm. The protein sample concentration was calculated so that samples loaded on to the gel would be of equal

concentration. The sample and sample buffer x 5 were added in the required volumes in a ratio of 1:4 and warmed for 5 min at 95°C. The samples were then loaded onto 12% gel SDS and run for an hour at 60 V, after that changed it to 100 V for 2 hours and transferred to a membrane. The membrane was stained with Ponceau S solution - Sigma to determine that the proteins had transferred onto the membrane. The membrane was washed with TBS-T and protein blocking was ensured by shaking for 1 hour, room temperature with 5% skim milk solution (Difco). The first antibody (for CTR1; GeneTex SLC314 in a ratio of 1:1000 and for Actin; DSHB JLA20 in a ratio of 1:500) was added and left in the shaker overnight at 4°C. Afterwards it was washed with TBS-T 3 times with 10 min of shaking between each wash. The second antibody was added for 1 hour at room temperature and then washed 3 times with TBS-T with 10 min of shaking between washes. For membrane development, the iBright FL1000 device was used with the Bio-Rad kit-Clarity western ECL substrate.

Raman Measurements

CuCysPhe solution (2.155 mM) was concentrated at Thermo Savant SPD11V SpeedVac Concentrator for 4 days at 36°C until a green crystal formed. Raman spectra of the crystals were collected at room temperature using an inVia Raman Microscope (Renishaw, United Kingdom) under an optical microscope, with a 50x objective. CuCysPhe, $CuCl_2$ and Cys peptide were measured using a 514 nm laser source and an 1800 gr/mm grating with a laser power of 0.5 mW, while the Cys peptide was excited at 10 mW. Due to its fluorescence, the phenanthroline was measured with a 785 nm laser using 0.25 mW excitation and a 1200 gr/mm grating.

Isothermal titration calorimetry (ITC)

The sample was first dialyzed against buffer A (50 mM Tris-HCl pH 8.0, 150 mM NaCl) supplemented with 10 mM ethylenediaminetetraacetic acid (EDTA) at 4°C overnight to remove any residual metal ions still bound to the protein and applied to a 5 mL desalting HiTrap column (Cytiva) to introduce the sample to buffer C (25 mM HEPES pH 7.4, 160 mM NaCl, 0.8 mM glycine). Sample concentration was quantified by UV light absorbance at 280 nm, using the theoretical molar extinction coefficient of 2980 $M^{-1}cm^{-1}$, further diluted to 100 µM in buffer C, filtered, degassed and slowly loaded into the isothermal cell of a MicroCal ITC200. ITC measurements were carried out at 25°C, (750 rpm stirring with 150 sec interval) with 15 consecutive injections of either 1 mM Cu(o-phe)(pep) or 1 mM $CuCl_2$, respectively dissolved in water or in buffer C. The heat effect during titration was calculated after subtraction of the dilution effect, and the dissociation constants were obtained by fitting the experimental data to a one-site binding model.

Computational Methods

System set-up

First, to derive force field parameters for investigated Cu-complexes (i.e., CuCysPhe, Cu^{2+}-Phe, Cu^{2+}-Cys, Cu^{+}-Phe, Cu^{+}-Cys and Cu-ATSM), we calculated force constants and Merz-Kollman restrained electrostatic potential (RESP) charges with Gaussian09 (Frisch) using the Metal Center Parameter Builder (MCPB) workflow (Li & Merz, 2016). POPC lipid bilayer was built with the CHARMM-GUI Membrane Builder (Wu et al., 2014). The lipid membrane was solvated with TIP3P water and K^+ and Cl^- ions were added at concentration of 0.15 M. The TLEAP tool from the AmberTools18 package was used to construct the topology using the Amber force field lipid14 (Maier et al., 2015) and Li and Merz 12 – 6 ion parameters (Li et al., 2015). Next, classical MD simulations were performed with GROMACS (version 2020.2) (Abraham et al., 2015) to relax the membrane-only system. After energy minimization, simulated systems were equilibrated in the NVT ensemble (T = 300 K) for 10 ns using periodic boundary conditions and position restraints on heavy atoms (1000 kJ/mol nm^2). Next, equilibration was continued in the NPT ensemble (p = 1 bar) without removing position restraints. After 10 ns, position restraints were limited to lipid head groups in the vertical (z) dimension and

equilibration was continued for another 10 ns. After, for each investigated Cu-complex, one instance of the molecule was added to the simulation box with packmol (Martinez et al., 2009). Before performing metadynamics, the obtained systems were simulated for 100 ns in the NPT ensemble without applying position restraints. Simulations were performed at 300 K and 1 bar. Temperature and pressure were monitored with Nose-Hoover thermostat (Bussi et al., 2007) (τ_T = 0.5 ps) and a Parrinello-Rahman barostat (Parrinello & Rahman, 1981) (τ_p = 2 ps), respectively. Newton's equations of motion were integrated with the leap-frog algorithm (2 fs time step) and electrostatic interactions were evaluated using the Particle Mesh Ewald method (Essmann et al., 1995).

Metadynamics

Well-tempered metadynamics simulations (Barducci et al., 2008) were performed in GROMACS (version 2020.2) patched with PLUMED (version 2.7.0) (Tribello et al., 2014). The distance between centre of mass of the POPC membrane and investigated Cu-complex along the vertical axis was used as a collective variable (CV). Gaussian hills with an initial height of 1.2 kJ mol^{-1} and bias factor equal to 8 were employed. The width of the Gaussian hills was 0.1 Å. A Gaussian hill was added to the potential energy surface every 500 steps. Simulations were performed for 500 ns. The free energy profile was calculated with the sum_hills utility.

Statistic

Statistical tests were performed using GraphPad Prism version 8.4.2 for Windows, GraphPad Software, Boston, MA, www.graphpad.com. Specific statistical tests are indicted for each experiment and statistical significance was set at *P < 0.05.

***In-vivo* experiments**

Animal experiments were performed according to the guidelines of the Animal Care and Use Committee of the Hebrew University (NIH approval number OPRR-A01-5011). Ethical Protocol MD-20-16223-4 submitted in March 2020. All mice were maintained in specific pathogen-free barrier facilities under a controlled 12 h light/12 h dark cycle and an ambient temperature of 21±1°C, humidity 40-50% at the Whol Institute for Translational Medicine, Hadassah Hospital. Sub-cutaneous tumors were induced by inoculation of $1{\cdot}10^6$ 4T1 (in 100µl) murine breast carcinoma cells into the lower back of female BALB/c OlaHsd mice (6-8 weeks old; Harlan, Ein Kerem, Israel). PET/MRI experiments were performed 7-10 days post-cancer cell injection.

In-vivo micro PET-MRI scanning

Experiments were performed at the Wohl Institute for Translational Medicine. PET-MRI images were acquired on a 7T 24 cm bore in a cryogen-free MR scanner based on the proprietary dry magnet technology (MR Solutions, Guildford, UK) with a 3-ring PET insert that uses the latest silicon photomultiplier (SiPM) technology (Courteau et al., 2021). The PET subsystem contains 24 detector heads arranged in three octagons of 116 mm in diameter. For MRI acquisition, a mouse quadrature RF volume coil was used. Mice were anesthetized with isoflurane vaporized with O_2. Isoflurane was used at 3.0% for induction and at 1.0-2.0% for maintenance. The mice were positioned on a heated bed, which allowed for continuous anesthesia and breathing rate monitoring. To determine the distribution of the radiotracers, the radioactive compounds (200 µl) were injected through a homemade small catheter inserted into the proximal tail vein. The ^{64}Cu-CysPhe (8.1±1MBq) or ^{64}Cu-ATSM (6.9±0.9 MBq) were administered outside the magnet. Two 20 min PET acquisitions were performed at 5 h and 24 h post-radiotracer injection. For comparison ^{18}F-FDG accumulation in 4T1 tumors was analyzed. The ^{18}F-FDG tracer (10±0.5 MBq) was i.v injected outside the magnet and 40 min later the mouse was positioned in the micro-PET/MRI scanner and scanned for 20 min scanning.

During PET acquisitions, MRI scans were acquired T1 & T2 weighted coronal spin echo images were collected for anatomical evaluation. Coronal T1-weighted images were acquired using the following parameters: TR= 1100 ms, TE=11 ms, echo spacing = 11 ms, FOV= 6x3 cm, slice thickness=1mm, 4 averages. Coronal T2-weighted images were acquired using the following parameters: TR= 4000 ms, TE=45 ms, echo spacing = 15 ms, FOV= 6x3 cm, slice thickness=1mm, and 4 averages. MRI and PET Images were analyzed using VivoQuant pre-clinical image post-processing software (Invicro). PET-MRI raw data were processed using the standard software provided by the manufacturers. PET data were acquired in list-mode, histogrammed by Fourier re-binning, and reconstructed using the 3D-OSEM algorithm, with standard corrections for random coincidences, system response, and physical decay applied. The reconstructed PET images from the PET/MR scanner were quantitated using a measured system-specific calibration factor to convert reconstructed count rates per voxel to activity concentrations (%ID/ml). Manual tissue segmentation of tumor, kidneys, liver, muscle, brain, and lung was carried out on co-registered 3D MR images. Regional ROIs were then used to calculate tissue radiotracer uptake from the reconstructed PET images. The images shown are coronal slices.

Ultrasound (US) and photoacoustic (PA) imaging

High-resolution ultrasound imaging was performed using a Vevo3100- LAZRX small animal US combined with a photoacoustic (PA) imaging system (Visualsonics, Toronto, Canada), with a MX-550D linear-array transducer (40-MHz center frequency) being used to acquire all images. The tunable laser supplied 10-20 mJ per pulse over the 680–970 nm wavelength range, with a pulse repetition frequency of 20 Hz. Once initialized, the system was switched to the oxy/hemo mode to measure sO_2 using the following parameters: depth, 10.00 mm; width, 14.08 mm; wavelength, 750 and 850 nm for the total hemoglobin concentration threshold (Hbt), and sO_2, respectively. Tumor sO_2 mapping was pseudo-colorized. Tumor baring mice underwent PA and Doppler examinations. For imaging, the mice were anesthetized with isoflurane (2.0%) and placed in a prone position on a heated platform, with body temperature, heart rate, and respiration rate monitored. All images were acquired by placing the probe directly over the tumor. Before sO_2 measurement, B-mode and Doppler US images were acquired to evaluate tumor vascularization and identify the tumor region of interest.

Immunohistochemistry (IHC)

Breast cancer tissue sections 40 µm in thickness were rinsed 5 times with PBS, containing 0.1% Triton X-100 (PBST) for 5 min. Non-specific binding were blocked with 10% normal horse serum (Vector Labs) + 2% BSA + 1% Glycine in PBST for 1 h at RT. Primary antibodies were diluted in antibody buffer (10% normal horse serum + 2% Bovine serum albumin (BSA) in PBST) (200 µl/well) and incubated overnight at 4°C. Anti-caspase-3 (Cell Signaling Technology (Asp175) (5A1E) 1:400), anti-HIF-1α (abcam [EPR16897] ab179483, 1:500) antibodies were used. Next, sections were rinsed five times in PBST for 5 min each time, and fluorescence-tagged secondary antibodies were applied for 1 h at RT. Slices were then stained with Hoechst 33342 (H3570 Invitrogen, Carlsbad, CA) diluted 1:1,000, followed by five 5 min rinses with PBST, and addition of mounting media and coverslips. Slides were photographed using a 20xwater objective for the Opera Phenix (PerkinElmer).

ITC measurements

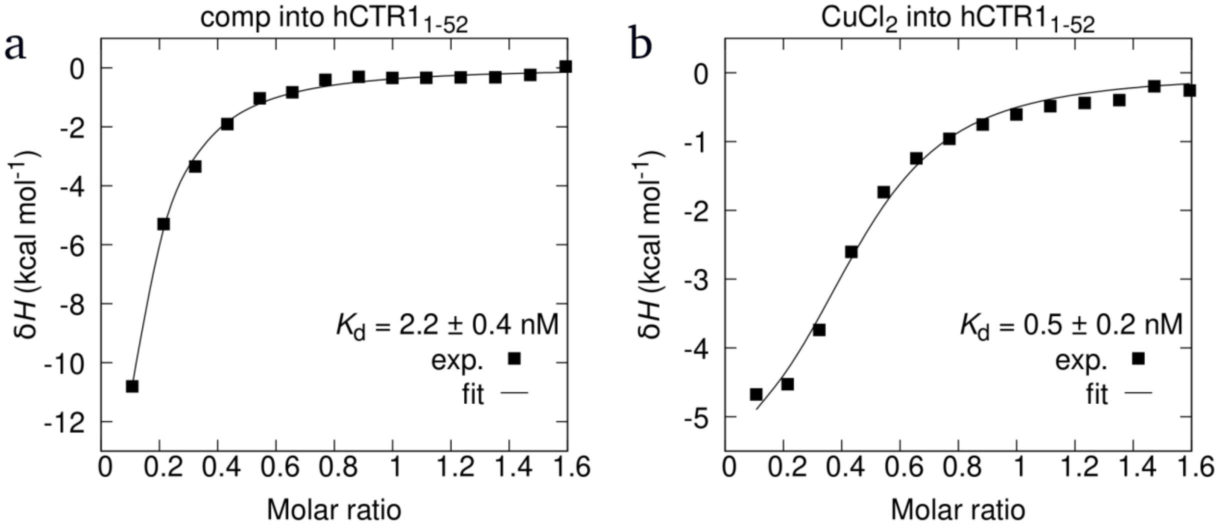

Figure S4: The interaction between Cu(o-phe)(pep) and the N-terminal domain of hCtr1 (hCtr$_{1-52}$) was studied by isothermal titration calorimetry (ITC). **a.** Heat effects during titration of hCtr$_{1-52}$ with Cu(o-phe)(pep) after subtraction of dilution effects. **b.** Heat effects during titration of hCtr$_{1-52}$ with $CuCl_2$. Dissociation constants were obtained by fitting a one-site binding model (black curve) to experimental data (black points). The results indicate that the affinity of Cu(o-phe)(pep) for the N-terminal domain is comparable to that of a naked Cu^{2+} ion, suggesting displacement of the peptide and/or ligand component by the N-terminal domain.

Data from cell experiments with non-radioactive Cu-CysPhe

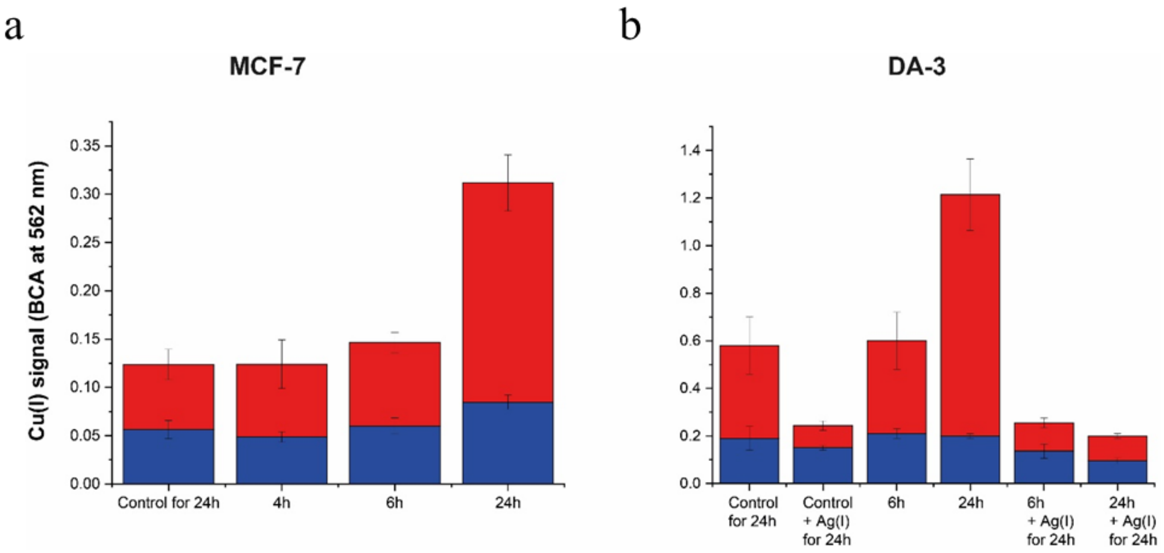

Figure S5: **Cu(I) signal in cells.** BCA absorption at 562 nm for the Cu-CysPhe complex in **a.** MCF-7 and **b.** DA-3 breast cancer cells. Ag(I) ions (50 μl of a 0.5 mM solution in water) were added together with the complex. The red color represents cells grown under hypoxic conditions, while blue indicates normoxic conditions.

Computational measurements

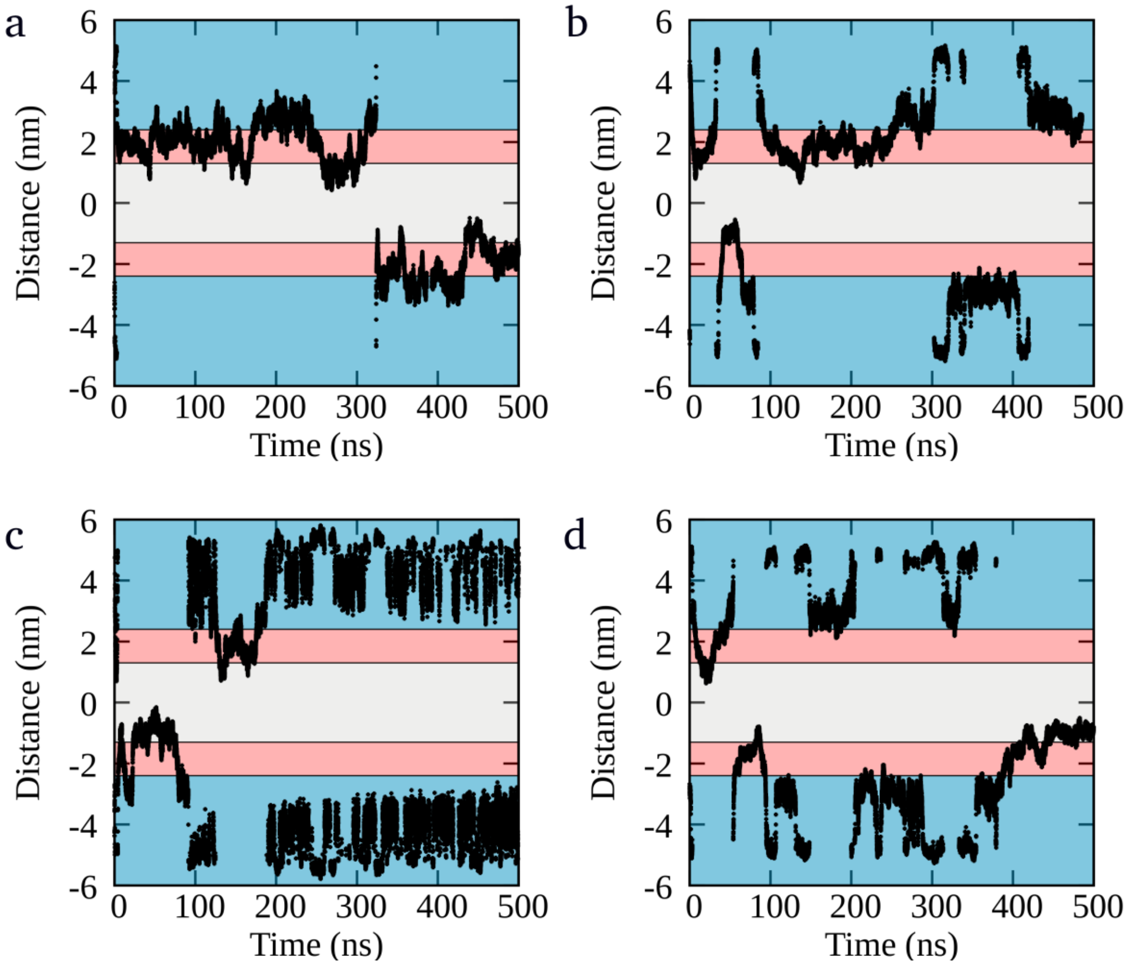

Figure S6. Distance from the center of the membrane along the Z axis for **a.** Cu^{2+}-ligand, **b.** Cu^{2+}-peptide, **c.** Cu^{+}-ligand and **d.** Cu^{+}-peptide system during metadynamics simulations. The area of the simulation box corresponding to aqueous solution is colored blue, while the polar heads and hydrophobic acyl chains of the lipid bilayer are marked in pink and gray respectively.

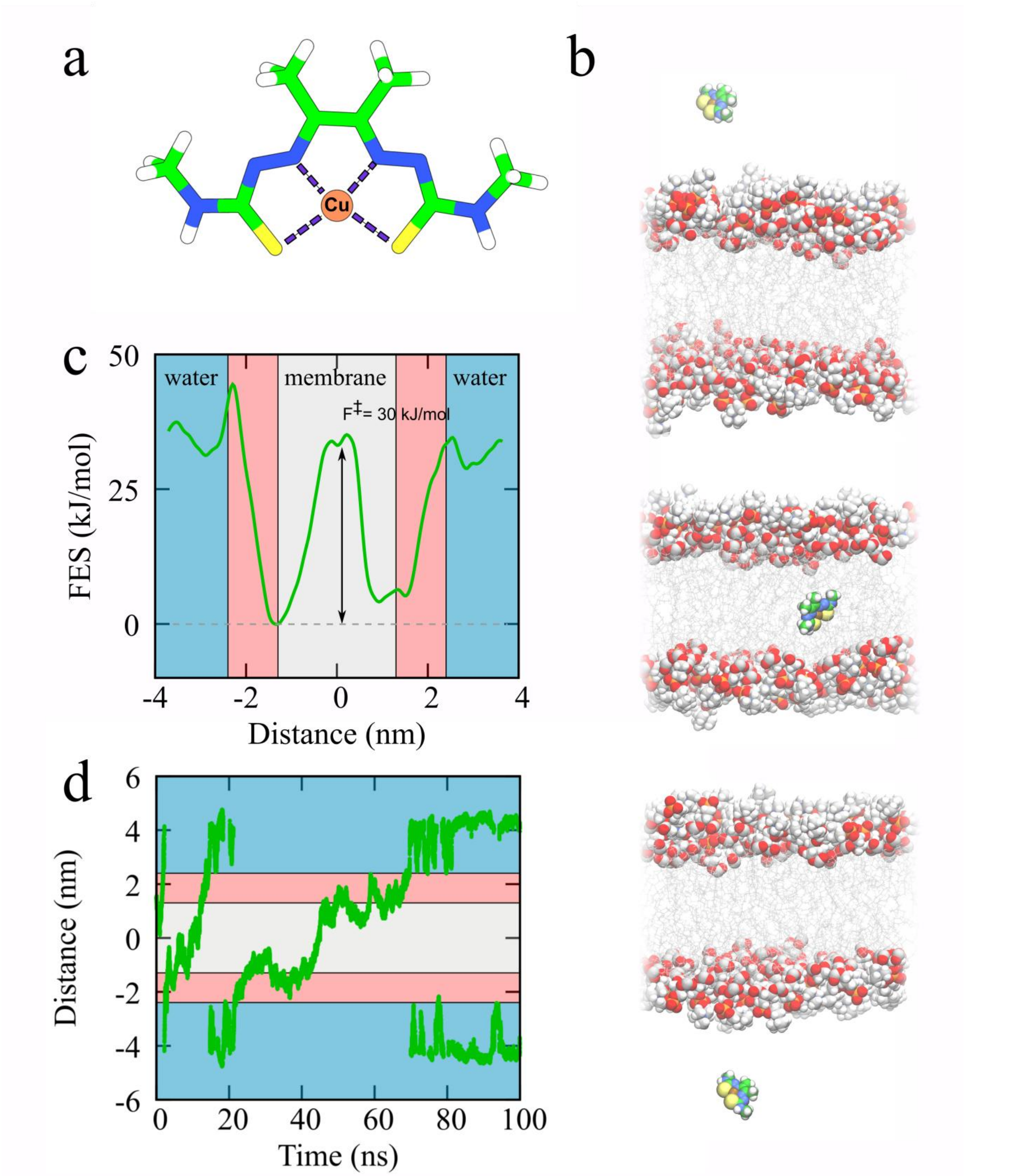

Figure S7. Analysis of membrane permeability of Cu-ATSM with metadynamics simulations. **a.** Scheme of Cu-ATSM. **b.** Simulation snapshots depicting one permeation event. The Cu-ATSM and lipid polar head groups are shown as van der Waals spheres, while hydrophobic lipid chains are shown as lines. **c.** One-dimensional free energy surface (FES) as a function of the distance between the center of mass of Cu-ATSM and the POPC membrane along the Z axis. The calculated free energy activation barrier ($F^{\ddagger}$) is 30 kJ/mol. The area of the simulation box corresponding to aqueous solution is coloured blue, while the polar heads and hydrophobic acyl chains of the lipid bilayer are marked in pink and grey, respectively. **d.** Distance between center of mass of the POPC membrane and Cu-ATSM along the Z axis. Several permeation events were observed for Cu-ATSM.

Molar activity

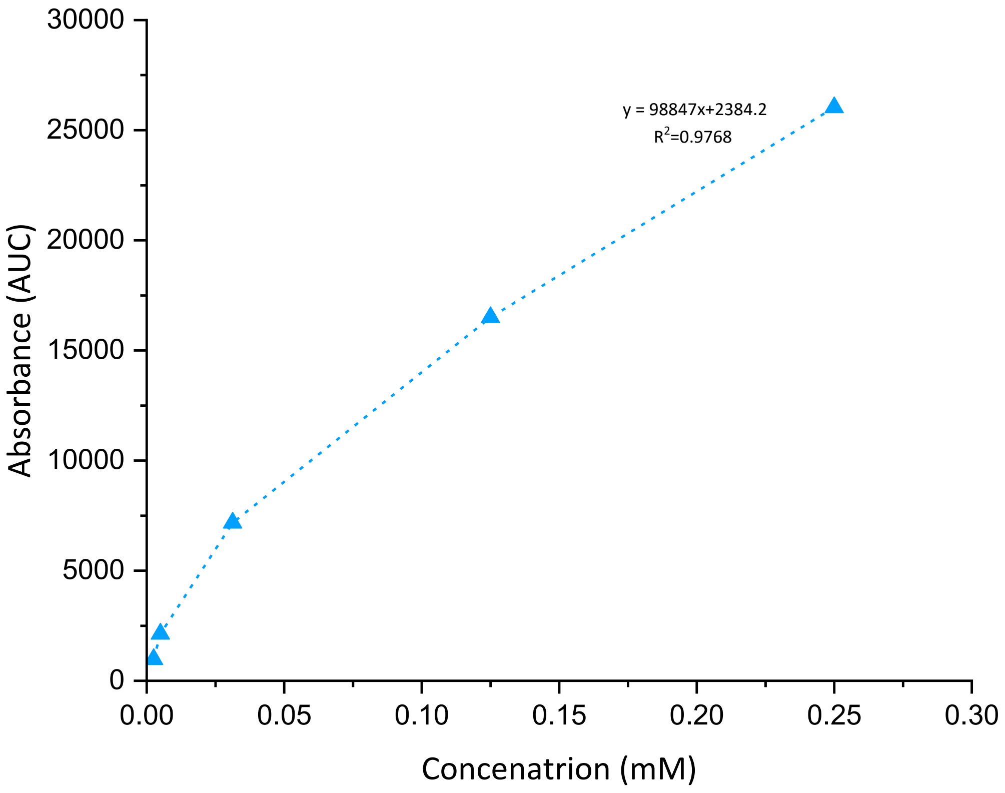

Figure S8: UV absorbance using HPLC for different complex concentrations.

Molar activity was calculated as follows:

UV absorbance of the non-radioactive complex was calculated at different complex concentrations. A linear curve was obtained where y represents the absorbance area and x is in mM. In the next step, various HPLC measurements were carried out on the radioactive complex. The area under the gamma absorption curve (termed here as A, and marked green in Fig. S9) was obtained. From the value of A, the x value was derived using the linear relation between the area and the concentration obtained above. The molar activity was then calculated using the following equation:

$$M.A. = \frac{activity\ of\ volume\ injected\ to\ the\ HPLC}{(injected\ volume\ ) \cdot x}\ [\frac{\mu Ci}{\mu mol}]$$

From six independent experiments, the molar activity was determined to be: 115.7 ± 3 μCi/μmol

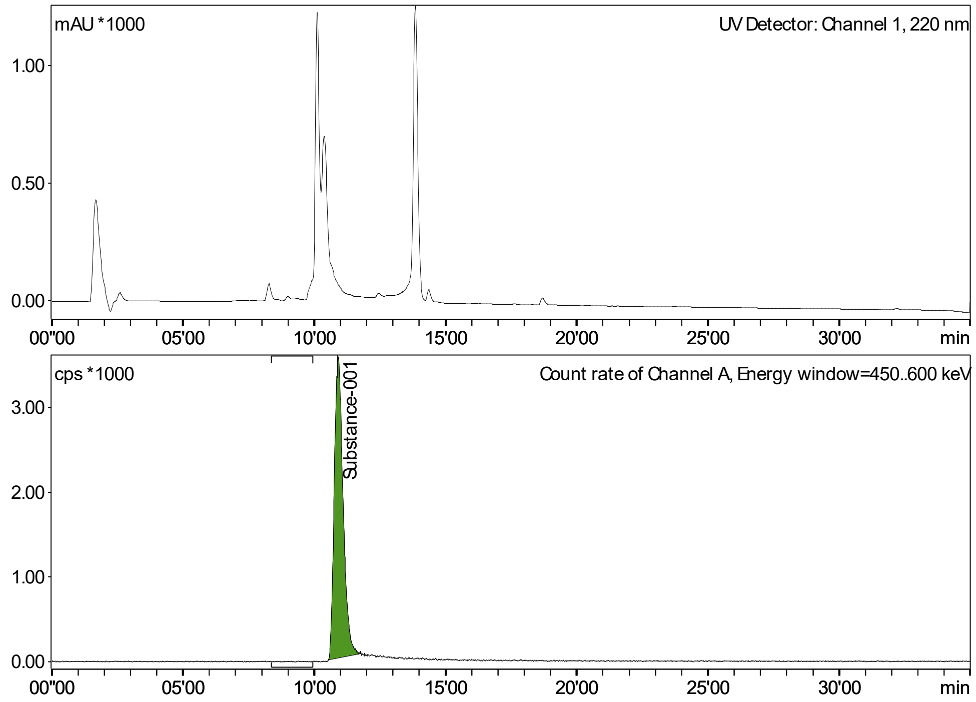

Figure S9: HPLC profiles of the ^{64}Cu-CysPhe complex using UV absorbance (top panel) and gamma radiation detection (bottom panel).

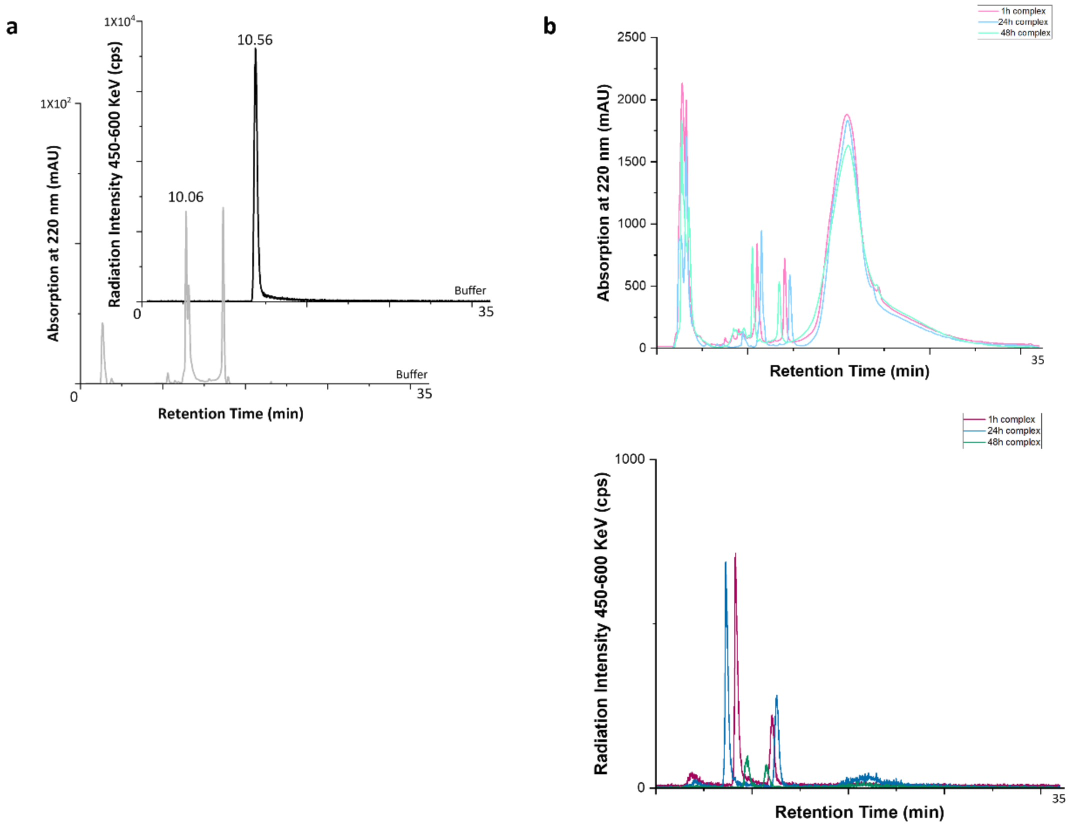

Figure S10: **a**. HPLC of ^{64}Cu-CysPhe in PBS buffer. **b**. HPLC of ^{64}Cu-CysPhe in Fetal bovine serum for 1, 24 and 48 hours.

Unprocessed Images of Western blots and corresponding gels

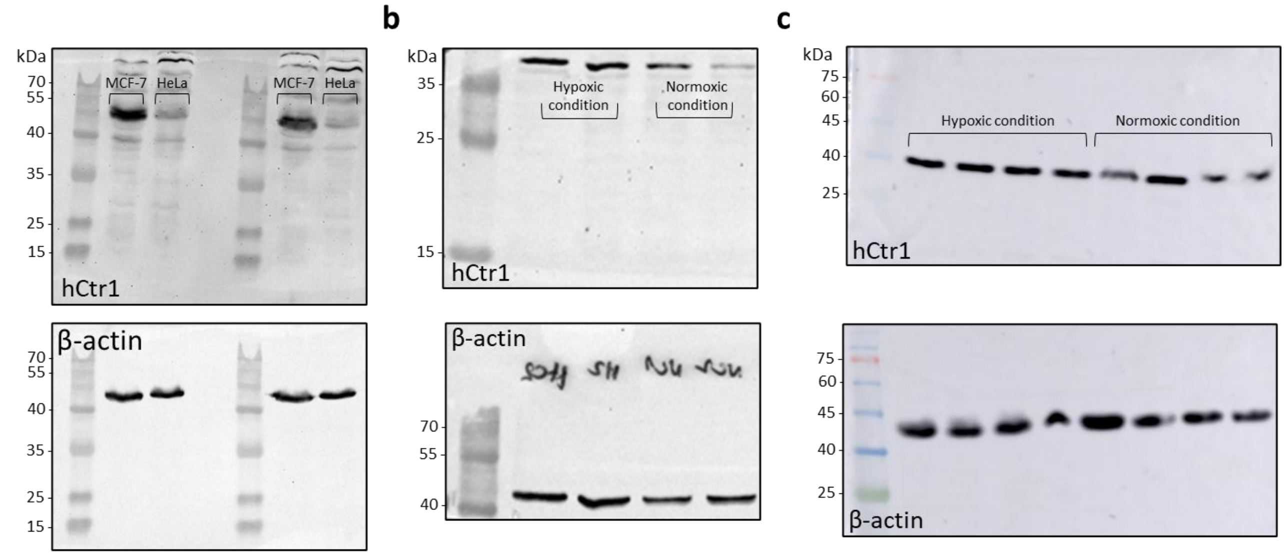

Figure S11: Western blot of hCtr1 and β-actin antibodies in **a**. MCF-7 and HeLa cells and **b**. MCF-7 treated for 24 hours under hypoxic and normoxic conditions. **c.** MCF-7 treated for 24 hours under hypoxic and normoxic conditions for the graph in figure 2a.

Data from cell experiments using radioactive ^{64}Cu-CysPhe in the presence of Ag(I) ions

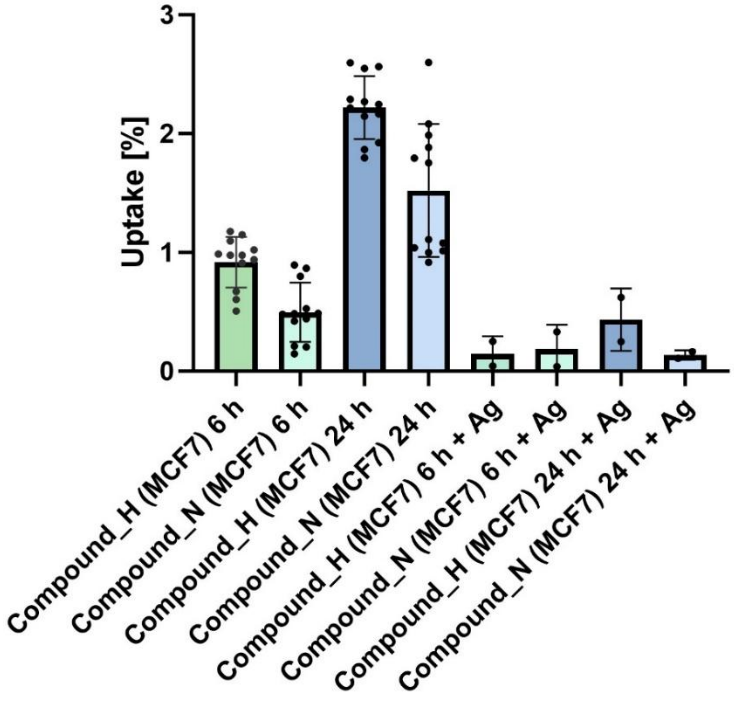

Figure S12: **Effect of Ag(I) ions.** Radioactive ^{64}Cu uptake by MCF-7 cells as a function of hypoxic (H) and normoxic (N) conditions and the presence of Ag(I) ions. Ag(I) ions (50 µl of a 0.5 mM solution in water) were added together with the complex.

Imaging PET-MRI data

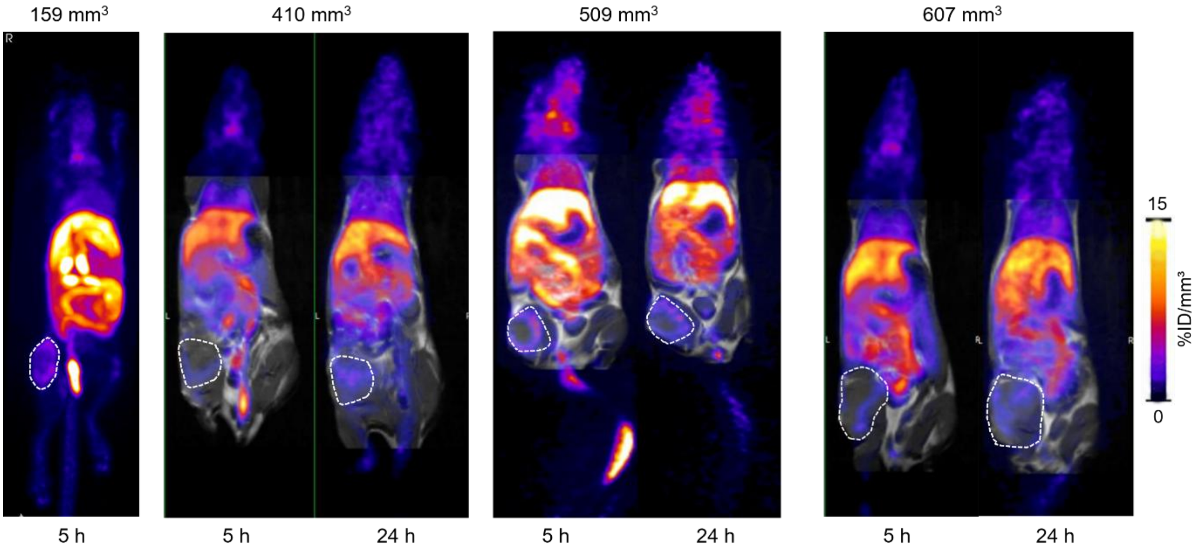

Figure S13: Micro PET-MRI experiments performed with the ^{64}Cu-CysPhe radiotracer. Tumor volume and imaging time after radiotracer injection are listed above and below each image respectively.

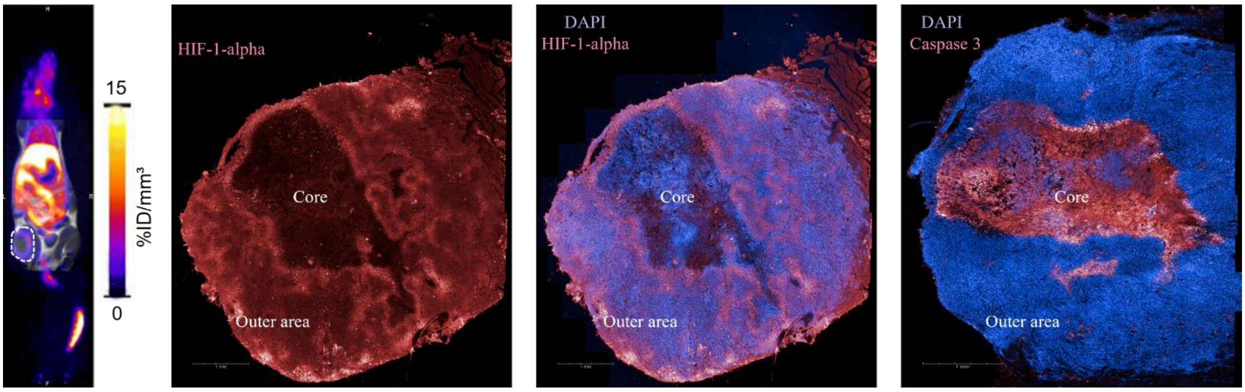

Figure S14: Immunohistochemical staining of consecutive sections breast cancer tissue from mice imaged in a PET/MRI. Expression of hypoxic and apoptotic area in tissues of a breast cancer tumor (363 ml) in a mouse.

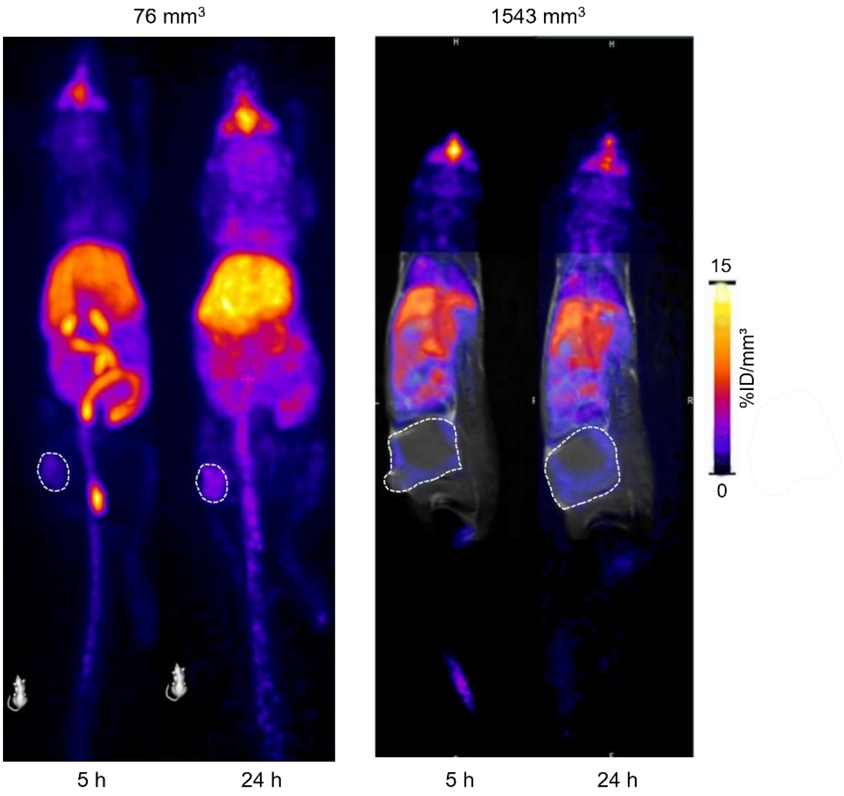

Figure S15: Micro PET-MRI experiments detected with the ^{64}Cu-ATSM radiotracer. Tumor volume and imaging time after radiotracer injection are listed above and below each image respectively.

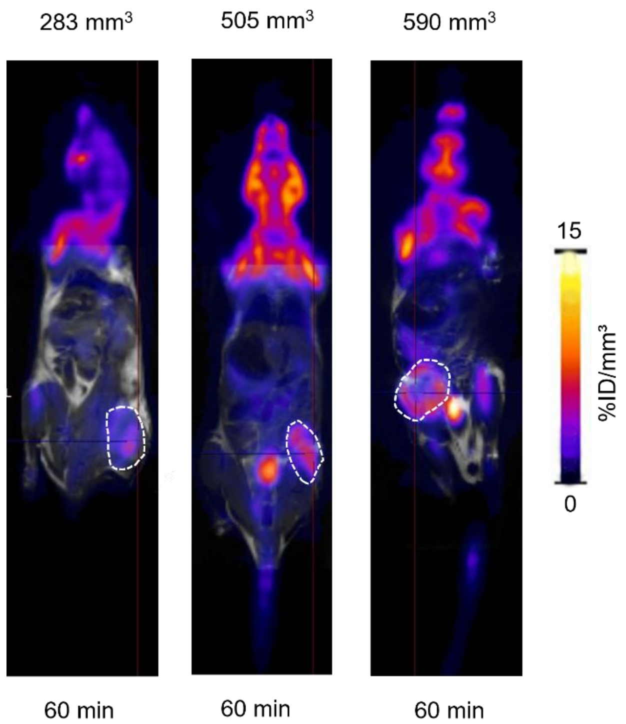

Figure S16: Micro PET-MRI experiments detected with the ^{18}F-FDG radiotracers. Tumor volume and imaging time after radiotracer injection are listed above and below each image respectively.

Data from 4T1 cell experiments using radioactive ^{64}Cu-CysPhe Vs ^{64}Cu-ATSM

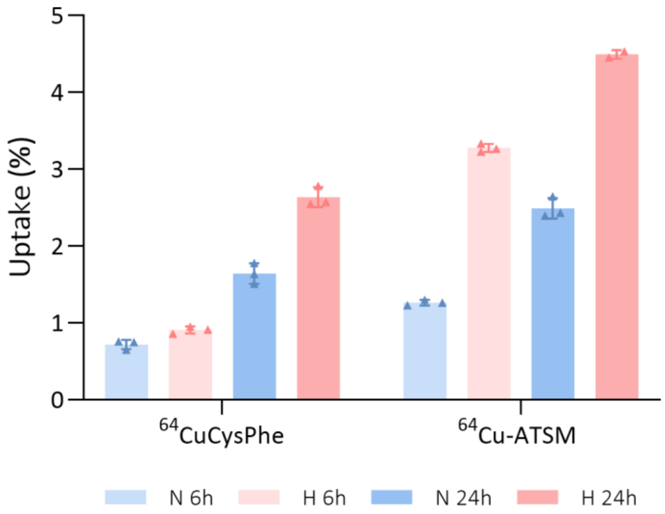

Figure S17: Radioactive ^{64}Cu-CysPhe Vs ^{64}Cu-ATSM uptake by 4T1 cells as a function of hypoxic (H) and normoxic (N) conditions for 6h and 24h.

References

Abraham, M. J., Murtaola, T., Schulz, R., Pall, S., Smith, J. C., Hess, B., & Lindahl, E. (2015). GROMACS: High performance molecular simulations through multi-level parallelism from laptops to supercomputers. *SoftwareX*, *1-2*, 19–25.

Barducci, A., Bussi, G., & Parrinello, M. (2008). Well-tempered metadynamics: A smoothly converging and tunable free-energy method. *Physical Review Letters*, *100*(2). https://doi.org/ARTN 020603

10.1103/PhysRevLett.100.020603

Bussi, G., Donadio, D., & Parrinello, M. (2007). Canonical sampling through velocity rescaling. *J Chem Phys*, *126*(1), 014101. https://doi.org/10.1063/1.2408420

Courteau, A., McGrath, J., Walker, P. M., Pegg, R., Martin, G., Garipov, R., Doughty, P., Cochet, A., Brunotte, F., & Vrigneaud, J. M. (2021). Performance Evaluation and Compatibility Studies of a Compact Preclinical Scanner for Simultaneous PET/MR Imaging at 7 Tesla. *IEEE Trans Med Imaging*, *40*(1), 205–217. https://doi.org/10.1109/TMI.2020.3024722

Essmann, U., Perera, L., Berkowitz, M. L., Darden, T., Lee, H., & Pedersen, L. G. (1995). A Smooth Particle Mesh Ewald Method. *Journal of Chemical Physics*, *103*(19), 8577–8593. https://doi.org/Doi 10.1063/1.470117

Frisch, M. J. e. a. Gaussian09 Revision E.01. (Gaussian, Inc. 2016). https://doi.org/https://gaussian.com/g09citation/

Gianelli, L., Amendola, V., Fabbrizzi, L., Pallavicini, P., & Mellerio, G. G. (2001). Investigation of reduction of Cu(II) complexes in positive-ion mode electrospray mass spectrometry. *Rapid Communications in Mass Spectrometry*, *15*(23), 2347–2353. https://doi.org/DOI 10.1002/rcm.510

Li, P., & Merz, K. M., Jr. (2016). MCPB.py: A Python Based Metal Center Parameter Builder. *J Chem Inf Model*, *56*(4), 599–604. https://doi.org/10.1021/acs.jcim.5b00674

Li, P., Song, L. F., & Merz, K. M., Jr. (2015). Systematic Parameterization of Monovalent Ions Employing the Nonbonded Model. *J Chem Theory Comput*, *11*(4), 1645–1657. https://doi.org/10.1021/ct500918t

Maccarrone, G., Caruso, R., Contino, A., Giuffrida, A., Messina, M., & Cucinotta, V. (2009). The Contribution of Electrospray Mass Spectrometry to the Study of Metal Complexes: The Case of Copper(II)-Dipeptide Systems. *European Journal of Inorganic Chemistry*(18), 2612–2620. https://doi.org/10.1002/ejic.200900086

Maier, J. A., Martinez, C., Kasavajhala, K., Wickstrom, L., Hauser, K. E., & Simmerling, C. (2015). ff14SB: Improving the Accuracy of Protein Side Chain and Backbone Parameters from ff99SB. *J Chem Theory Comput*, *11*(8), 3696–3713. https://doi.org/10.1021/acs.jctc.5b00255

Martinez, L., Andrade, R., Birgin, E. G., & Martinez, J. M. (2009). PACKMOL: a package for building initial configurations for molecular dynamics simulations. *J Comput Chem*, *30*(13), 2157–2164. https://doi.org/10.1002/jcc.21224

Parrinello, M., & Rahman, A. (1981). Polymorphic Transitions in Single-Crystals - a New Molecular-Dynamics Method. *Journal of Applied Physics*, *52*(12), 7182–7190. https://doi.org/Doi 10.1063/1.328693

Peisach, J., & Blumberg, W. E. (1974). Structural implications derived from the analysis of electron paramagnetic resonance spectra of natural and artificial copper proteins. *Arch Biochem Biophys*, *165*(2), 691–708. https://doi.org/10.1016/0003-9861(74)90298-7

Plonka, D., Kotuniak, R., Dabrowska, K., & Bal, W. (2021). Electrospray-Induced Mass Spectrometry Is Not Suitable for Determination of Peptidic Cu(II) Complexes. *J Am Soc Mass Spectrom*, *32*(12), 2766–2776. https://doi.org/10.1021/jasms.1c00206

Stoll, S., & Schweiger, A. (2006). EasySpin, a comprehensive software package for spectral simulation and analysis in EPR. *J Magn Reson*, *178*(1), 42–55. https://doi.org/10.1016/j.jmr.2005.08.013

Tribello, G. A., Bonomi, M., Branduardi, D., Camilloni, C., & Bussi, G. (2014). PLUMED 2: New feathers for an old bird. *Computer Physics Communications*, *185*(2), 604–613. https://doi.org/10.1016/j.cpc.2013.09.018

Walke, G., Aupic, J., Kashoua, H., Janos, P., Meron, S., Shenberger, Y., Qasem, Z., Gevorkyan-Airapetov, L., Magistrato, A., & Ruthstein, S. (2022). Dynamical interplay between the human high-affinity copper transporter hCtr1 and its cognate metal ion. *Biophys J*, *121*(7), 1194–1204. https://doi.org/10.1016/j.bpj.2022.02.033

Walke, G. R., Meron, S., Shenberger, Y., Gevorkyan-Airapetov, L., & Ruthstein, S. (2021). Cellular Uptake of the ATSM-Cu(II) Complex under Hypoxic Conditions. *ChemistryOpen*, *10*(4), 486–492. https://doi.org/10.1002/open.202100044

Walke, G. R., & Ruthstein, S. (2019). Does the ATSM-Cu(II) Biomarker Integrate into the Human Cellular Copper Cycle? *ACS Omega*, *4*(7), 12278–12285. https://doi.org/10.1021/acsomega.9b01748

Wu, E. L., Cheng, X., Jo, S., Rui, H., Song, K. C., Davila-Contreras, E. M., Qi, Y., Lee, J., Monje-Galvan, V., Venable, R. M., Klauda, J. B., & Im, W. (2014). CHARMM-GUI Membrane Builder toward realistic biological membrane simulations. *J Comput Chem*, *35*(27), 1997–2004. https://doi.org/10.1002/jcc.23702
